# Genomic, Proteomic and Morphological Characterization of Two Novel Broad Host Lytic Bacteriophages ΦPD10.3 and ΦPD23.1 Infecting Pectinolytic *Pectobacterium* spp. and *Dickeya* spp.

**DOI:** 10.1371/journal.pone.0119812

**Published:** 2015-03-24

**Authors:** Robert Czajkowski, Zofia Ozymko, Victor de Jager, Joanna Siwinska, Anna Smolarska, Adam Ossowicki, Magdalena Narajczyk, Ewa Lojkowska

**Affiliations:** 1 Laboratory of Plant Protection and Biotechnology, Department of Biotechnology, Intercollegiate Faculty of Biotechnology, University of Gdansk and Medical University of Gdansk, Gdansk, Poland; 2 Netherlands Institute of Ecology (NIOO-KNAW), Wageningen, the Netherlands; 3 Laboratory of Biological Plant Protection, Department of Biotechnology, Intercollegiate Faculty of Biotechnology, University of Gdansk and Medical University of Gdansk, Gdansk, Poland; 4 Laboratory of Electron Microscopy, Faculty of Biology, University of Gdansk, Gdansk, Poland; ContraFect Corporation, UNITED STATES

## Abstract

Pectinolytic *Pectobacterium* spp. and *Dickeya* spp. are necrotrophic bacterial pathogens of many important crops, including potato, worldwide. This study reports on the isolation and characterization of broad host lytic bacteriophages able to infect the dominant *Pectobacterium* spp. and *Dickeya* spp. affecting potato in Europe *viz*. *Pectobacterium carotovorum* subsp. *carotovorum* (Pcc), *P*. *wasabiae* (Pwa) and *Dickeya solani* (Dso) with the objective to assess their potential as biological disease control agents. Two lytic bacteriophages infecting stains of Pcc, Pwa and Dso were isolated from potato samples collected from two potato fields in central Poland. The ΦPD10.3 and ΦPD23.1 phages have morphology similar to other members of the *Myoviridae* family and the *Caudovirales* order, with a head diameter of 85 and 86 nm and length of tails of 117 and 121 nm, respectively. They were characterized for optimal multiplicity of infection, the rate of adsorption to the Pcc, Pwa and Dso cells, the latent period and the burst size. The phages were genotypically characterized with RAPD-PCR and RFLP techniques. The structural proteomes of both phages were obtained by fractionation of phage proteins by SDS-PAGE. Phage protein identification was performed by liquid chromatography-mass spectrometry (LC-MS) analysis. Pulsed-field gel electrophoresis (PFGE), genome sequencing and comparative genome analysis were used to gain knowledge of the length, organization and function of the ΦPD10.3 and ΦPD23.1 genomes. The potential use of ΦPD10.3 and ΦPD23.1 phages for the biocontrol of *Pectobacterium* spp. and *Dickeya* spp. infections in potato is discussed.

## Introduction

Soft rot *Enterobacteriaceae* (SRE): *Pectobacterium* spp. and *Dickeya* spp. are ubiquitous necrotrophic bacterial pathogens of agriculturally important crops worldwide; they can be isolated from plants, soil, and water as well as from the surface of and within insects [1]. Due to the fact that SRE can seriously affect plant health and crop yield and because they are widely spread in the environment, these bacteria are ranked among the top ten most significant bacterial pathogens in agriculture [2].

In potato, the main soft rot *Enterobacteriaceae* causing blackleg, which affects the growing plant, and tuber soft rot of potato in storage and transit are *Pectobacterium carotovorum* subsp. *carotovorum* (Pcc), *Pectobacterium atrosepticum* (Pba) [3], *P*. *carotovorum* subsp. *brasiliense* (Pcb) [4], *P*. *wasabiae* (Pwa) [5] and several *Dickeya* spp. among which *D*. *solani* (Dso) has been recognized recently as the most important [5–7]. In Europe, blackleg and soft rot disease may result in relatively high losses in potato crops not only in the field and in storage but also indirectly in seed potato production due to declassification and rejection of seed lots [8].

In temperate climatic zones, the symptoms caused by the different SRE in potato are often difficult to discriminate [6, 9]. SRE bacteria are able not only to establish a systematic infection but also ultimately to kill the host plant [7, 9]. They characteristically produce large quantities of different extracellular plant cell wall degrading enzymes (e. g. cellulases, pectinases, proteases, pectate lyases, polygalacturonases, pectin methylesterases phospholipases) that allow the bacteria to infiltrate vascular compartments and result in a maceration (rotting) of plant host tissues [2, 6, 9].

Of all *Pectobacterium* spp. infecting potato, Pcc has the widest host range globally, whereas Pba is associated mainly with potato grown under temperate climate [10]. Recently, a highly aggressive bacterium Pcb was found to cause symptoms in potato in tropical and temperate regions [4, 11] and Pwa found for the first time in horse radish in Japan [5, 12] and was later on potato in New Zeeland, South Africa, Canada and several European countries [13, 14]. In contrast, *Dickeya* spp. can affect a number of plant species in different temperature zones [6], however, until recently it has been associated with blackleg and tuber soft rot only in tropical and subtropical regions. Until 2005, in temperate climates *Dickeya* spp. were considered of less importance and only weakly virulent strains of *D*. *dianthicola* were isolated from blackleg diseased plants in Western and Northern Europe [15]. However, due to the recent introduction of a new virulent *Dickeya* species—Dso—to the potato ecosystem [16], the situation has changed and now infection of potato by Dso is dominant in Europe, leading to even higher economic losses than in the past [17, 18].

Since the prohibition of mercury-based compounds as a pesticide and the general disapproval in Europe of antibiotics utilization in agriculture, attempts to control SRE in potato have been ineffective due to the lack of suitable tools and strategies [8]. Currently, the management of the SRE in potato is therefore based mainly on hygienic practices during plant cultivation and tuber storage as well as on seed certification based on the use of healthy (axenic) initial propagative material in seed production, limited number of generations and rejection of highly infected crops during multiplication [8]. So far, these measures have been partially successful but have not led to the total control of soft rot and blackleg pathogens [6, 19].

Bacteriophages (phages) have been proposed as potential biological control agents against plant pathogenic bacteria. They have been evaluated for different pathogens e. g. *Erwinia amylovora*, *Xanthomonas pruni*, *Pseudomonas tolaasii*, *Streptomyces scabies* and *Ralstonia solanacearum* (for review see [20]). Phages were also experimentally tested to control *Pectobacterium* spp. [21] and *Dickeya* spp. [22, 23] in potato and other crops with relative success but only limited attempts have been made to characterize these lytic bacteriophages [22–24].

Despite numerous potential benefits of bacteriophage-based biological control of plant pathogenic bacteria (for review see [25, 26]), few attempts have been made to test their efficacy by direct treatment of plants or planting material in large scale greenhouse or field experiments. One important difficulty is their well-known narrow host specificity [27]. Usually a particular bacteriophage is able to infect and kill only a limited range of strains of any one bacterial species [28]. This narrow specificity may hinder the value of bacteriophages in agriculture especially in situations in which more than one closely related pathogen is present. *Pectobacterium* spp. and *Dickeya* spp. can coexist in infected plant tissues [29–31], although there is little information on the relative importance of any synergistic effect in disease development [32]. It has been suggested that bacteria from both genera may interact in plants to facilitate infection [31, 33]. To our knowledge, there are no reports describing broad spectrum host lytic bacteriophages able to infect members of several species of soft rot *Enterobacteriaceae*.

The aim of this study was to isolate and characterize in detail broad host range lytic bacteriophages able to infect members of *Pectobacterium* spp. and *Dickeya* spp. especially against dominant soft rot and blackleg pathogens of potato in Europe *viz*. Pcc, Pwa and *D*. *solani*.

## Materials and Methods

### Bacterial strains and media

*Pectobacterium* and *Dickeya* spp. isolates used in this study are listed in S1 Table. For routine tests, bacteria were grown at 28°C for 24–48 h on tryptone soya agar (TSA; Oxoid) or nutrient agar (NA; Oxoid) prior to use, unless stated otherwise. For liquid preparations, bacterial cultures were grown in tryptone soya broth (TSB; Oxoid) or in nutrient broth (NB; Oxoid) at 28°C with agitation at 200 rpm. For long-term storage, bacterial cultures were kept in sterile 40% (v/v) glycerol at −80°C.

### Isolation of bacteriophages from environmental samples and enrichment of bacteriophages in their host bacterial cultures

Soil, rhizosphere soil and potato plant and tuber samples were obtained from the Main Inspectorate of Plant Health and Seed Inspection, Poland. One hundred sixty four samples were collected between April 2013 and September 2013 in different regions in Poland. To isolate the bacteriophages from the environment the procedure described before [22] was used. *D*. *solani* strain IPO2222 [16], Pcc strain Ecc71 [34], Pba strain SCRI 1043 [35], Pwa strain SCC3193 [36], Pcb strain LMG 21371 [4] and *D*. *dianthicola* strain CFBP 1200 [37] were used to enrich lytic bacteriophages from soil samples as described previously [38]. After incubation, bacteria were removed by centrifugation (8000 ×*g* for 5 min) and supernatants were filter-sterilized with 0.22 μm syringe filters.

### Purification of individual phage particles

Purification of single phage particles was done using a soft top agar method [39] with several modifications [22]. Plaques obtained after the fourth purification were collected, resuspended in 2 ml sterile Ringer’s buffer (Merck), shaken for 30 min at 200 rpm to release viral particles, filter-sterilized with a 0.22 μm membrane filter (VWR) and stored for further analysis at 4°C. Phage densities (plaque-forming units (pfu) ml^-1^) were calculated for each bacteriophage.

### Bacteriophage morphology under transmission electron microscopy (TEM)

Transmission electron microscopy analysis of phage particles was performed in the Laboratory of Electron Microscopy, Faculty of Biology, University of Gdansk, Gdansk, Poland using negative staining of phage particles with uranyl acetate as described previously [22].

### Determination of the bacteriophages’ host range

A host specificity assay was performed using 99 bacterial isolates: 41 isolates of *Dickeya* spp. belonging to six genomo-species [37] (eight isolates of *D*. *dadantii*, six isolates of *D*. *dianthicola*, five isolates of *D*. *zeae*, two isolates of *D*. *paradisiaca*, four isolates of *D*. *chrysanthemi*, 16 isolates of ‘*D*. *solani’*) and 58 isolates of five *Pectobacterium* species and subspecies [3] (25 isolates of *P*. *atrosepticum*, 24 isolates of *P*. *carotovorum* subsp. *carotovorum*, 7 isolates of *P*. *wasabiae* and 2 isolates of *P*. *carotovorum* subsp. *brasiliense*) (S1 Table) as previously described before [22].

### Determination of the optimal multiplicity of infection (MOI)

Optimal MOI, defined as the optimal ratio between phage particles and bacterial host cells [40], was determined for phages ϕPD10.3 and ϕPD23.1. Bacterial culture (IPO2222, Ecc71 or SCC3193) was infected with phages at four different pfu/cfu ratios (MOI): 0.01, 0.1, 1 and 10. After an overnight incubation at 28°C with shaking (200 rpm), bacterial cultures were centrifuged (10 000 ×*g*, 10 min) and supernatants were assayed for phage titre as described above. The MOI resulting in the highest phage titre (the highest pfu ml^-1^) was considered as optimal. The experiment was repeated three times and the results from all repetitions were averaged.

### Phage adsorption to host bacterial cells

To determine the speed of phage adsorption to bacterial host cells, 1 ml of log-phase *Dickeya* spp. IPO2222, Pcc Ecc71 or Pwa SCC3193 cells (10^8^ cfu ml^-1^) was infected with a phage suspension (ϕPD10.3 or ϕPD23.1) to reach an MOI of 0.1 (*ca*. 10^7^ pfu ml^-1^) and incubated at 28°C for up to 20 min. After 0, 1, 2, 5, 10 and 20 min, two individual samples per phage were collected and centrifuged at 10 000 ×*g* for 5 min to sediment the bacteria together with the adsorbed bacteriophages. The resulting supernatants were filter-sterilized with a 0.22 μm syringe filter and assayed for free, unadsorbed phages. The experiment was repeated three times and the results were averaged. Phage adsorption was calculated as follows: percentage adsorption = ((control titre − residual titre) / control titre) × 100 [22].

### One-step growth

To determine the latent period and the burst size of the ϕPD10.3 and ϕPD23.1 bacteriophages in bacterial hosts IPO2222, Ecc71 and SCC3193, a one-step growth experiment was conducted [41] with some modifications as described previously [22]. The number of phage particles was determined by the soft top agar method as described above. Viable bacterial counts were determined both before the bacteria were mixed with phages and at the end of the experiment. Burst size was estimated from three independent experiments (six independent measurements) by using the following equation: burst size = ΔV/ΔB, where ΔV—indicates changes in the number of phage particles and ΔB—represents the changes in the bacterial number during the experiment as described earlier [42].

### Estimation of bacteriophage genome size by Pulsed-Field Gel Electrophoresis (PFGE)

PFGE analysis of intact phage particles was prepared as previously described [43] with slight modifications. Briefly, 400 μl of purified phage suspensions (*ca*. 10^14^ pfu ml^-1^; in PS buffer −0.1 M Tris, 0.1 M EDTA, pH = 8.0) were transferred to 2 ml-microcentrifuge tubes preheated to 50°C in a thermoblock and mixed with 400 μl of molten 1.2% plug agarose (1.2% SeaKem Gold Agarose (Cambrex Corp.) in 1×TE buffer). 250 μl of samples was transferred to wells of plug casting mold allowing them to solidify at room temperature for *ca*. 30 min. After the plugs had solidified, they were transferred to the 15 ml tubes (Sarstedt) containing 5 ml of PL buffer (50 mM Tris, 50 mM EDTA, 1% SDS) and 25 μl of proteinase K (Thermo Scientific, 20 mg ml^-1^) and incubated at 54°C for 2 h to lyse phage capsids. After lysis, the plugs were washed four times with sterile TE buffer for 10 min. Prepared agar plugs containing the intact phage DNA were stored at 4°C in sterile TE buffer before analysis. Phage DNA was run without digestion in a 1% SeaKem Gold Agarose gel in 0.5×TBE (45 mM Tris-borate, 1 ml EDTA, pH = 8.0) containing 100 mM thiourea (Sigma) with a CHEF-DR III chiller system (Bio-Rad Laboratories Inc.) using 0.5×TBE buffer with 100 mM thiourea as a running buffer. PFGE was performed at 6 V/cm field strength for 22 h at 14°C with the pulse time linearly increased from 2.2 to 54.2 s and a fixed reorientation angle of 120°. The gels were stained with 5 mg ml^-1^ ethidium bromide (Sigma), washed with water and analyzed under UV light in GelDoc (Biorad) imaging system. *Salmonella enterica* serotype Braenderup (strain H9812, ATCC) was used as a DNA marker [44].

### Purification of bacteriophage genomic DNA for RFLP, RAPD-PCR and whole genome sequencing

To obtain high phage titer (*ca*. 10^13^–10^14^ pfu ml^-1^) for genomic DNA purification, the ϕPD10.3 and ϕPD23.1 phages were enriched in *D*. *solani* IPO2222 culture as described previously [22]. After enrichment, bacterial cells were removed by centrifugation (8000 ×*g*, 20 min) and the resulting phage suspension (*ca*. 50 ml) was filtered through a 0.22 μm membrane filter (VWR) to remove bacterial debris. Phage suspension was treated with DNase I (Sigma-Aldrich) (final concentration: 0.5 mg ml^-1^) for 60 min at 37°C with shaking (100 rpm) to digest the bacterial DNA. Phage particles were further purified and concentrated *via* centrifugation (15 000 ×g, 4°C) for 2 h. The supernatant was then removed and the pellet containing bacteriophage particles was resuspended in 500 μl of 5 mM MgSO_4_ and washed two times with 5 mM MgSO_4_ under the same conditions [45]. The phage genomic DNA was isolated as described previously [46].

### Restriction fragment length polymorphism (RFLP) of phage genomes

Purified phage genomic DNA (*ca*. 200–400 ng μl^-1^) was subjected to a single-enzyme restriction analysis with *Nco*I, *Nde*I *Bam*HI, *Hind*III, *Kpn*I, *Sal*I, *Alu*I, *Xba*I, *Eco*RI, *Ksp*AI, *Alu*I, *Rsa*I, *Hpa*II and *Hin*6I (ThermoScientific) restriction endonucleases (RE) according to the manufacturer’s protocol. Briefly, phage genomic DNA (*ca*. 200 ng per reaction) was digested for up to 24 h with 2.5 U of a single restriction endonuclease in separate digestions in a total volume of 10 μl. Digested DNA was electrophoresed in 1–2% agarose (0.5×TBE) gels. For the estimation of the size of the DNA fragments, λ genomic DNA digested with *Hind*III and *Eco*RI was used. Agarose gels were stained with 5 mg ml^-1^ of GelRed (Biotium) for visualization of DNA.

### RAPD-PCR of phage genomes

The RAPD-PCR was executed according to Comeau *et al*. (2004) using primer R10D (5’-GTCASSWSSW-3’, where S and W represent G/C and A/T, respectively) [47] with the following modifications. The phage DNA concentration was adjusted with Milipore water (MQ) to a final concentration of approximately 100 ng μl^-1^. RAPD was performed in a total volume of 50 μl containing 1.5 U Platinum Taq DNA polymerase (Invitrogen), 4.5 mM MgCl_2_, 0.6 mM of each deoxytriribonucleoside triphosphate (dNTPs), 2 μM of the R10D primer and 2–10 μl of phage DNA. PCR was carried out with the following program: initial denaturation at 95°C for 1.5 min., followed by 40 cycles of denaturation at 95°C for 45 s, annealing at 40°C for 3 min, extension at 72°C for 1 min and a final extension at 72°C for 10 min. As a control, instead of phage DNA, host bacterial DNA (*ca*. 100 ng μl^-1^) was used. The amplified DNA was analyzed by electrophoresis in a 1.5% agarose gel in 0.5×TBE buffer stained with 5 mg ml^-1^ of ethidium bromide (Sigma). Gels were run for *ca*. 8 h at 100 V and at the room temperature (approx. 20–24°C). A 1 kb and 100 bp ladders (Fermentas) were used as size markers.

### Phage genomes sequencing, annotation and *in silico* comparative analysis

Phage ϕPD10.3 and ϕPD23.1 genomic DNA were sequenced using the Illumina technology and *de novo* re-assembled at Baseclear, The Netherlands (www.baseclear.com) using CLC Main Workbench program (http://www.clcbio.com/products/clc-main-workbench/). Structural and functional annotations were obtained from the IGS Annotation Service (Institute for Genome Sciences, University of Maryland School of Medicine automated pipeline http://ae.igs.umaryland.edu/cgi/index.cgi) and from RAST (Rapid Annotation using Subsystem Technology, accessed *via* the http://rast.nmpdr.org/ website). Data for phage genome assembly were subsampled from the Illumina paired reads. Fractions of 0.01, 0.03, 0.04, 0.05, 0.06, 0.07, 0.08, 0.09, 0.10, 0.20, 0.25, 0.33 0.50 were sampled from the reads with three different seeds (s10, s20, s30) using the tool seqtk (seqtk sample-s10 <file> <fraction>) (https://github.com/lh3/seqtk). The sampled fractions were assembled using Mira 4.9.3 (http://sourceforge.net/p/mira-assembler/wiki/Home/) and examined for complete phage contigs. The phage genomes were mapped and annotated using available genomic sequences deposited in GenBank (http://www.ncbi.nlm.nih.gov/genbank/). The *in silico* analysis of bacteriophages’ genomes was done using Manatee (http://manatee.sourceforge.net/) accessed *via* website of Institute for Genome Sciences, University of Maryland School of Medicine. The lifestyle of phages ϕPD10.3 and ϕPD23.1 (temperate or lytic) was predicted using PHACTS [48]. Multiple genome alignment was performed using Mauve [49] and comparative genomics analyses were done using EDGAR [50]. We used a complete genome sequence of *Enterobacteriaceae* bacteriophage T4 (GenBank accession number: NC_000866.4) as outgroup for comparative bacteriophage genome analyses. To find potential genes acquired by the ϕPD10.3 and ϕPD23.1 coding for toxins and allergens bacteriophage genomes were subjected to bioinformatic analysis using Virulence Finder 1.2 (http://cge.cbs.dtu.dk/services/VirulenceFinder/) and VirulentPred (http://203.92.44.117/virulent/submit.html) accessed *via* the given websites.

### Analyses of bacteriophage ϕPD10.3 and ϕPD23.1 proteins

#### SDS-PAGE analysis of phage proteins

For SDS-PAGE electrophoresis, phages ϕPD10.3 and ϕPD23.1 (*ca*. 10^10^ pfu ml^-1^) were diluted two times with sterile demineralized water. Laemmli buffer (4% SDS, 20% glycerol, 10% 2-mercaptoethanol, 0.004% bromophenol blue and 0.125 M Tris-HCl, pH 6.8, 6 × concentrated stock) was added to the samples to obtain 2 × concentrated working solution and the mixture was firstly frozen in liquid nitrogen for 1–2 min and then boiled for 5 min at 95°C. Twenty five μl of phage extracts were separated in 12% acrylamide SDS-PAGE gel (Rothiphorese Gel 30, 37.5:1) (ROTH) for 19 h at 50 V at room temperature (22°C). PageRuler Pre-stained Protein Ladder (Thermo Scientific), prepared according to instructions provided by the manufacturer, was used as a size marker. The gel was stained with PageBlue Coomasie Blue (Thermo Scientific) for 18 h and destained with sterile demineralized water for 6 h at room temperature (22°C) as suggested by the manufacturer. Protein bands were excised from the gel with a sterile scalpel and used for mass spectrometry analysis.

#### Mass spectrometry analysis of the phage ϕPD10.3 and ϕPD23.1 proteins

Electrospray ionization MS/MS analysis of phage proteins was performed at Mass Spectrometry Laboratory, Institute of Biochemistry and Biophysics, Polish Academy of Sciences in Warsaw, Poland.

#### Structural/functional assignment of unknown phage proteins based on their 3D protein structures

In order to predict the molecular functions of the unknown structural proteins obtained from the SDS-PAGE and MS analysis of phage ϕPD10.3 and ϕPD23.1 proteins we used GeneSillico Protein Structure Prediction Meta-server (https://genesilico.pl/meta2/acl_users) containing known three-dimensional (3D) protein structures [51] and PSI-BLAST accessed *via* website of NCBI (http://www.ncbi.nlm.nih.gov/) [52]. The predicted proteins with the highest scores were considered as the most valid [51, 52].

### Effect of bacteriophages on potato tuber tissue maceration caused by *D*. *solani* IPO2222, *P*. *wasabiae SCC*3139 and *P*. *carotovorum* subsp. *carotovorum* Ecc71

#### A potato slice assay

A potato slice assay was used to assess if the ϕPD10.3 and ϕPD23.1 bacteriophages were able to protect potato tuber tissue when co-inoculated on potato slices with a mix of Dso IPO2222, Pcc Ecc71 and Pwa SCC3193 [22]. The bacteriophages’ concentration was adjusted to 10^5^ pfu ml^-1^ in sterile demineralized water and the bacterial density was adjusted to 10^7^ cfu ml^-1^ in demineralized sterile water for each strain. Ware potato tubers of cultivar Bryza, obtained locally were surface-sterilized with 70% ethanol for 10 min, rinsed with tap water and dried with tissue paper. Potato tubers were cut into *ca*. 0.7 cm-thick transverse disks, using a sterile knife. For each slice, three wells (5 × 5 × 5 mm) were made using a sterile cork borer and these were filled with 50 μl of a mixture of containing 10^5^ pfu ml^-1^ of one of the tested bacteriophages together with 10^7^ cfu ml^-1^ of each bacterial strain tested (IPO2222 + Ecc71 + SCC3193). Three potato slices obtained from three different potato tubers were used per treatment. As a negative control, 50 μl aliquots of sterile demineralized water was used instead of bacterial and phage suspensions, while as a positive control, 50 μl of bacterial suspension containing 10^7^ cfu ml^-1^ of three bacterial strains (IPO2222 + Ecc71 + SCC3193) was used. The protective effect of the phages on the potato tissue was measured after incubation for 72 h at 28°C in a humid box by calculating the ratio of the average diameter of rotten potato tissue around the wells co-inoculated with bacteria and bacteriophage to the average diameter of rotten tissue around wells inoculated with bacterial mixture only. Two duplications were made for each test and the entire experiment was independently repeated two times with the same setup. Results from the experiments were averaged.

#### A whole potato tuber assay

Whole potato tuber assay [23] was used to check whether application of ϕPD10.3 and ϕPD23.1 would protect potato tubers from vascular infections caused by a mix of Dso IPO2222, Pcc Ecc71 and Pwa SCC3193. Briefly, the bacteriophage and bacteria were prepared in the exact same way as described above for the potato slice assay. Ware potato tubers of cultivar Bryza, were surface-sterilized with 70% ethanol for 10 min, rinsed with tap water and dried with tissue paper. All tubers were weighted before the experiment. Per tuber, one 0.5 cm-thick transverse slice taken at a tuber rose end (end opposite to the stolon end) was incised and the cap was removed. 100 μl of the mixture containing 10^7^ cfu ml^-1^ of each bacterial strain tested (IPO2222 + Ecc71 + SCC3193) in sterile demineralized water was pipetted on the surface of the capless tuber. Inoculated tubers were left for 30 min. allowing the bacteria to be absorbed into the (vascular) tuber tissue. As a control, 100 μl of sterile demineralized water was used instead of bacterial suspensions. To evaluate the protection effect of ϕPD10.3 and ϕPD23.1, 100 μl of phage suspension containing 10^5^ pfu ml^-1^ of either individual or mixture of both phages was added onto the surface of infected or control tubers and these were once more left until the suspensions were absorbed inside vascular tissue. As a control, instead of bacteriophage suspensions, 100 μl of sterile demineralized water was used. The cut cap was re-attached on each tuber with a sterile toothpick. Treated tubers were left for 72 h at 28°C in a humid box. Ten potato tubers were used per treatment and the experiment was repeated two times with the same setup. The weight of the rotting tissue was measured for each tuber. The protective effect of the phage on the potato tubers was measured by calculating the percentage of rotting mass of each tuber. The results were averaged per treatment and per experiment.

## Results

### Isolation of bacteriophages

Between April and September 2013, 164 samples of potato rhizosphere soil, bulk soil, potato stem and tubers collected from arable potato fields in different regions in Poland were screened for the presence of lytic bacteriophages against different *Pectobacterium* and *Dickeya* species. After enrichment of potential bacteriophages in bacterial host cultures, 28 (17% of all tested samples) samples yielded lytic bacteriophages able to kill at least one of the six strains of bacterial species tested (*P*. *carotovorum* subsp. *carotovorum*, *P*. *wasabiae*, *P*. *atrosepticum*, *P*. *carotovorum* subsp. *brasiliense*, *D*. *solani* and *D*. *dianthicola*). From each positive sample one distinct plaque was isolated and further purified to obtain pure phage particles (Table 1). Enrichment of bacteriophages in their host bacterial cells resulted in phage suspensions with high titre averaging from 10^12^ to 10^14^ pfu ml^-1^ after an overnight incubation at 28°C.

**Table 1:** Bacteriophages isolated in this study.

No.	phage	Plague description ^a^	Sample origin ^b^	Geographical region in Poland
**1**	ϕPD 2.1	small, transparent	potato tuber	Warminsko-Mazurskie (Warmian-Masurian Province)
**2**	ϕPD 2.2	medium, semitransparent	bulk soil	Warminsko-Mazurskie (Warmian-Masurian Province)
**3**	ϕPD 3.1	big, transparent	bulk soil	Pomorskie (Pomeranian Province)
**4**	ϕPD 4.4	small, transparent	bulk soil	Lubelskie (Lublin Province)
**5**	ϕPD 4.6	medium, transparent	rhizosphere soil	Pomorskie (Pomeranian Province)
**6**	ϕPD 5.2	big, semitransparent	potato tuber	Mazowieckie (Masovian Province)
**7**	ϕPD 5.4	big, transparent	potato tuber	Podlaskie (Podlaskie Province)
**8**	ϕPD 7.1	big, transparent	bulk soil	Kujawsko-Pomorskie (Kuyavian-Pomeranian Province)
**9**	ϕPD 8.1	big, transparent	rhizosphere soil	Mazowieckie (Masovian Province)
**10**	ϕPD 8.5	medium	rhizosphere soil	Mazowieckie (Masovian Province)
**11**	ϕPD 9.1	big, transparent	potato tuber	Pomorskie (Pomeranian Province)
**12**	ϕPD 10.3	medium, transparent	potato stem	Mazowieckie (Masovian Province)
**13**	ϕPD 11.3	big, transparent	bulk soil	Mazowieckie (Masovian Province)
**14**	ϕPD 11.4	medium, semitransparent	bulk soil	Wielkopolskie (Greater Poland Province)
**15**	ϕPD 12.6	small, transparent	potato tuber	Pomorskie (Pomeranian Province)
**16**	ϕPD 15.3	small, transparent	bulk soil	Mazowieckie (Masovian Province)
**17**	ϕPD 17.1	big, transparent	potato stem	Pomorskie (Pomeranian Province)
**18**	ϕPD 18.2	small, transparent	potato stem	Pomorskie (Pomeranian Province)
**19**	ϕPD 20.2	medium, semitransparent	rhizosphere soil	Mazowieckie (Masovian Province)
**20**	ϕPD 22.1	big, transparent	bulk soil	Wielkopolskie (Greater Poland Province)
**21**	ϕPD 23.1	big, transparent	potato tuber	Wielkopolskie (Greater Poland Province)
**22**	ϕPD 25.1	big, transparent	potato tuber	Mazowieckie (Masovian Province)
**23**	ϕPD 26.2	medium, semitransparent	rhizosphere soil	Mazowieckie (Masovian Province)
**24**	ϕPD 27.2	medium, transparent	potato stem	Kujawsko-Pomorskie (Kuyavian-Pomeranian Province)
**25**	ϕPD 31.1	big, transparent	rhizosphere soil	Mazowieckie (Masovian Province)
**26**	ϕPD 31.2	small, transparent	potato tuber	Mazowieckie (Masovian Province)
**27**	ϕPD 32.3	big, semitransparent	potato tuber	Lubelskie (Lublin Province)
**28**	ϕPD 33.3	small, semitransparent	rhizosphere soil	Pomorskie (Pomeranian Province)

^a^—plague formation and plague characteristics were evaluated on lawns of *Pectobacterium carotovorum* subsp. *carotovorum* Ecc71 (bacteriophages’ host) after 24 h incubation at 28°C, plagues were characterized according to their diameter (*small*: diameter 0–2.0 mm, *medium*: diameter 2.1–4,0 mm, *big*: diameter 4.1 mm and wider) and transparency (*transparent*: clear halo plague and *semitransparent*: imperfectly transparent plague)

^b^—164 samples of soil (bulk soil), soil adhering to the potato (*Solanum tuberosum* L.) roots (rhizosphere soil), tuber and potato stem samples were collected from various arable potato fields in Poland and tested for presence of lytic bacteriophages against soft rot *Enterobacteriaceae* (*D*. *solani* strain IPO2222, *P*. *carotovorum* subsp. *carotovorum* strain Ecc71, *P*. *atrosepticum* strain SCRI 1043, *P*. *wasabiae* strain 3193, *P*. *carotovorum* subsp. *brasiliense* strain LMG 21371 and *D*. *dianthicola* strain CFBP 1200)

### Bacteriophage host range

In order to find bacteriophages able to infect more than one species or subspecies of SRE, the above described 28 bacteriophages were tested for to lyse 99 *Dickeya* and *Pectobacterium* spp. strains including the type strains and environmental isolates; 8 isolates of *D*. *dadantii*, 6 isolates of *D*. *dianthicola*, 5 isolates of *D*. *zeae*, 2 isolates of *D*. *paradisiaca*, 4 isolates of *D*. *chrysanthemi*, 16 isolates of *D*. *solani*, 25 isolates of *P*. *atrosepticum*, 24 isolates of *P*. *carotovorum* subsp. *carotovorum*, 7 isolates of *P*. *wasabiae* and 2 isolates of *P*. *carotovorum* subsp. *brasiliense* were used (S1 Table). Of all bacteriophages tested, only two named ϕPD10.3 and ϕPD23.1 (abbreviations: ϕ—phage, P—*Pectobacterium*, D—*Dickeya*, number reflects the sample number) possessed the broadest host range from all the phages isolated in this study (were able to infect strains of Pcc, Pwa and Dso isolates) (S1 Table). Consequently, these two were selected for further analysis. None of the bacteriophage isolates tested in this study was able to infect strains of the remaining species (and subspecies) of soft rot *Enterobacteriaceae*, including Pba (S1 Table).

### Transmission electron microscopy (TEM) analysis

**Fig 1:**
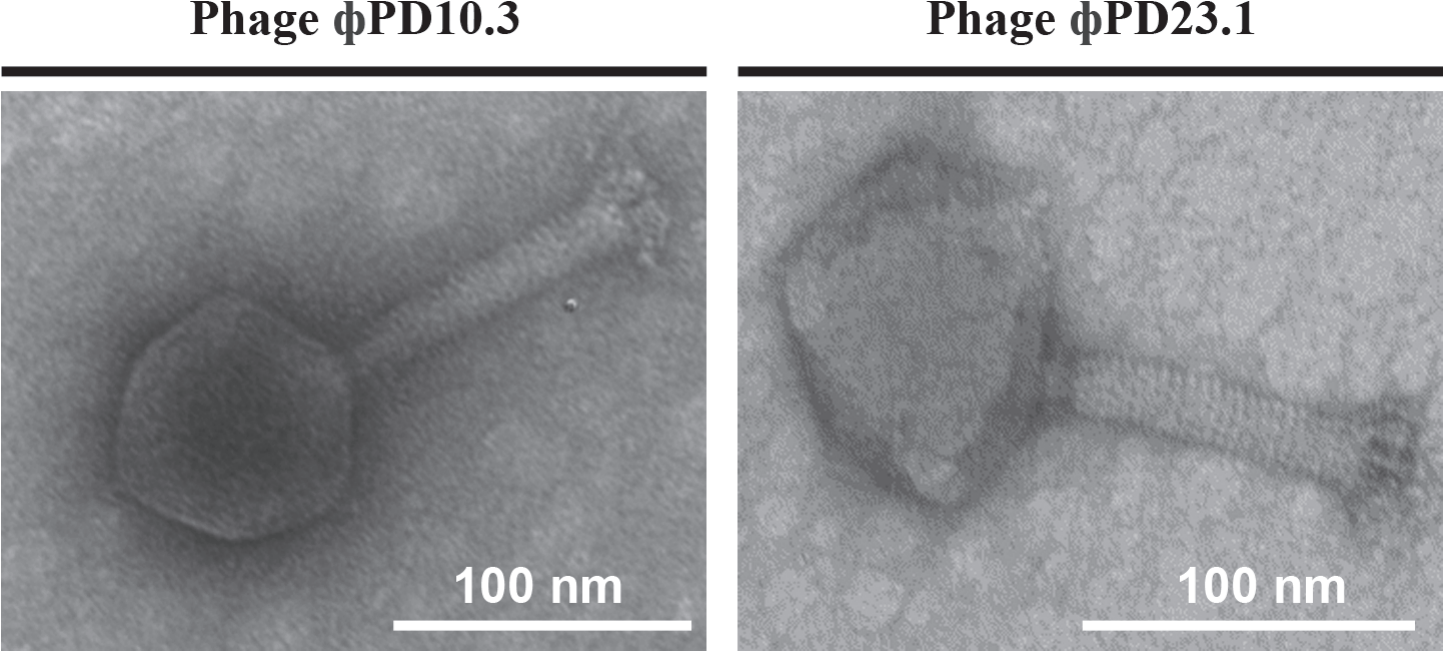
Transmission electron micrographs (TEM) of *Pectobacterium* spp. and *Dickeya* spp. bacteriophages ϕPD10.3 and ϕPD23.1 stained with uranyl acetate. For TEM, bacteriophages were purified four times by passaging individual plaques using soft top agar and IPO2222, Ecc71 and SCC 3193 as hosts. Phage suspensions of *ca*. 10^14^–10^18^ pfu ml^-1^ in 5 mM MgSO_4_ were used. Each photograph represents a typical bacteriophage particle. At least 10 photographs were taken of 10 different bacteriophage particles for each sample and preparation. Bar represents 100 nm.

Transmission electron microscopy analysis performed for ϕPD10.3 and ϕPD23.1 revealed that both phages belonged to the order *Caudovirales*, family *Myoviridae* based on their morphology and presence of icosahedral head, neck and contractile tail (Fig. 1). The diameter of the heads was 85 and 86 nm, and the length of the tails was 117 nm and 121 nm for phages ϕPD10.3 and ϕPD23.1, respectively.

### Phage adsorption to host bacterial cells, optimal MOI, latent period and burst size

In order to better understand the interaction of bacteriophages with their bacterial hosts, ϕPD10.3 and ϕPD23.1 were assessed for their adsorption study to hosts, estimation of the optimal MOI and in one-step growth experiments in which the latent period and the burst size were measured. Optimal MOI, adsorption, latent period and burst size differed greatly for ϕPD10.3 and ϕPD23.1, for a given bacterial host. In general, for both phages, the optimal MOI was between 0.01 and 0.1, adsorption at 28°C after 20 min was between 70 and 83% and burst size was between 82 and 102 phages per host cell (Table 2).

**Table 2:** Interaction of bacteriophages ϕPD10.3 and ϕPD23.1 with their bacterial hosts *D*. *solani* IPO2222, *P*. *carotovorum* subsp. *carotovorum* Ecc71 and *P*. *wasabiae* 3193.

Bacteriophage	Bacterial host ^a^	Optimal MOI ^b^	Adsorption ^c^	Latent period ^d^	Burst size ^e^
ϕ**PD10.3**	IPO 2222	0.01	70%	30 min.	95 ± 7
ϕ**PD10.3**	Ecc71	0.1	75%	40 min.	82 ± 5
ϕ**PD10.3**	3193	0.1	76%	30 min.	102 ± 3
ϕ**PD23.1**	IPO2222	0.01	74%	30 min.	95 ± 5
ϕ**PD23.1**	Ecc 71	0.1	83%	40 min.	91 ± 6
ϕ**PD23.1**	3193	0.01	71%	20 min.	95 ± 5

^a—^Three bacterial hosts were used for the analysis; *D*. *solani* IPO2222, *P*. *carotovorum* subsp. *carotovorum* Ecc71 and *P*. *wasabiae* 3193

^b—^Optimal MOI (optimal multiplicity of infection) defined as the optimal ratio between phage particles (pfu) and bacterial host cells (cfu) was determined using three bacterial hosts (IPO2222, Ecc71 and 3193) for phages ϕPD10.3 and ϕPD23.1. Bacterial culture was infected with phages at four different pfu/cfu ratios (MOI): 0.01, 0.1, 1 and 10. The MOI resulting in the highest phage titre was considered as the optimal one. The experiment was independently repeated three times with the same setup and the results from all repetitions were averaged.

^c^—Adsorption of bacteriophages to bacterial cells was measured at 28°C *in vitro* for 20 min. For testing the speed of phage ϕPD10.3 and ϕPD23.1 adsorption to the bacterial host cells 1 ml of log-phase IPO2222, Ecc71 or 3193 10^8^ colony forming units ml^-1^ was infected with a phage suspension to reach MOI 0.1. After incubation for 0 (control), 1, 2, 5, 10 and 20 min, two individual samples per phage were collected and centrifuged at 10000 g for 5 min to sediment the bacteria together with the adsorbed bacteriophages. The resulting supernatants were filter sterilized with a 0.22 μm syringe filter and assayed for free, unadsorbed phages. The experiment was independently repeated three times.

^d^—Latent period defined as a time between phage adsorption to the bacterial cell and burst (lysis of host cell) was calculated from one-step growth experiment using 3 bacterial hosts (IPO2222, Ecc71 or 3193). For this, the ϕPD10.3 or ϕPD23.1 phages were allowed to absorb to bacterial cells for 20 min at 28°C. After 20 min. the suspension was diluted 10 000 times in TSB prewarmed to 28°C and incubated with shaking (ca. 200 rpm) at 28°C. Two samples of 100 μl were taken per phage and bacterial host tested, every 10 min. over a period of 100 min. The number of phage particles was determined by the soft top agar method as described above. Bacterial viable counts were determined both before bacteria were mixed with phages and at the end of the experiment.

^e^—Burst size was estimated from three independent experiments (six independent measurements) by using the following equation: burst size = ΔV/ΔB, where ΔV represents changes in the number of phage particles and ΔB represents the changes in the bacterial number during the experiment

### RFLP, RAPD-PCR and PFGE analysis

#### RFLP analysis

**Fig 2:**
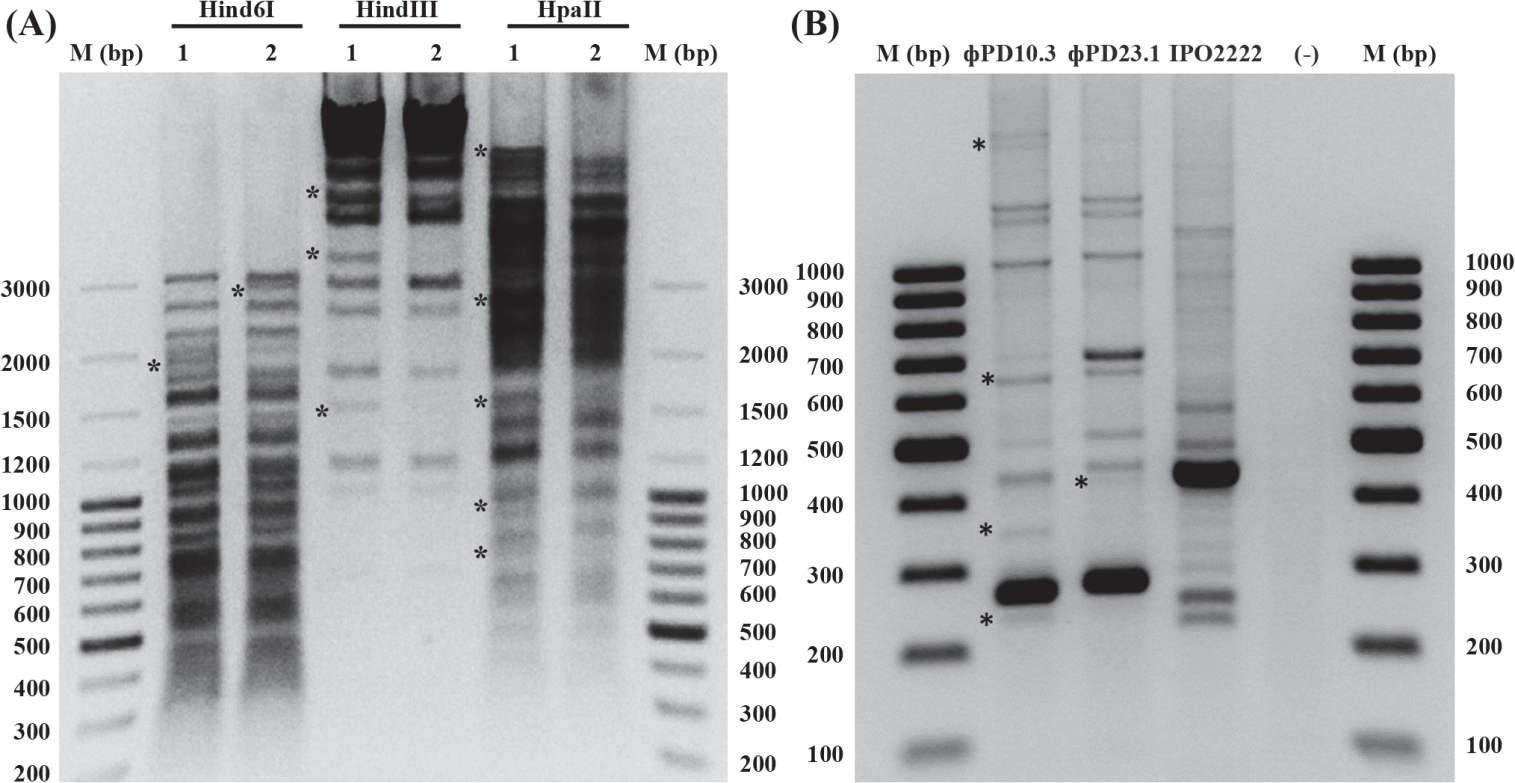
Fingerprinting of ϕPD10.3 and ϕPD23.1 bacteriophage genomic DNA with the use of restriction fragment length polymorphism (RFLP) (A) and Random Amplified Polymorphic DNA-PCR (RAPD-PCR) (B) analysis. (A) Digestion of bacteriophage genomic DNA of ϕPD10.3 and ϕPD23.1 phages with endonucleases *Hin*6I, *Hind*III and *Hpa*II (all from Fermentas). Phage DNA (*ca*. 200–400 ng ml^-1^) was digested with 2.5 U of individual restriction endonuclease in single digestions for up to 24 h at 37°C according to protocol provided by the manufacturer. The digested DNA was ran on 1 or 1.5% agarose gel in 0.5 x TBE for up to 3 h and visualized under UV light by staining with 0.5 mg ml^-1^ GelRed. GeneRuler 100 bp Plus DNA ladder (Fermentas) was used as a size marker. The asterisks (*) indicate DNA bands present either in ϕPD10.3 or ϕPD23.1 only. (B) RAPD-PCR band patterns obtained for ϕPD10.3 and ϕPD23.1 bacteriophages and *D*. *solani* IPO2222 (bacteriophage host) using ϕPD10.3 and ϕPD23.1 and IPO2222 genomic DNA as templates and primer R10D. The RAPD patterns were run on 1 or 1.5% agarose gel in 0.5 x TBE for up to 5 h and were visualized under UV light by staining with 0.5 mg ml^-1^ GelRed. GeneRuler 100 bp DNA ladder (Fermentas) was used as a size marker. The asterisks (*) indicate bands present either in ϕPD10.3 or ϕPD23.1 only. For negative control (-), RAPD-PCR was performed with the same protocol, however, instead of DNA template an equal volume of sterile demineralized water was added to the reaction mixture.

Phage ϕPD10.3 and ϕPD23.1 genomic DNA samples were insensitive to digestion with a majority of the restriction endonucleases tested (*Nco*I, *Nde*I, *Bam*HI, *Kpn*I, *Sal*I, *Alu*I, *Xba*I, *Eco*RI, *Ksp*AI, *Alu*I, *Rsa*I) except *Hin*6I, *Hind*III and *Hpa*II. The ϕPD10.3 and ϕPD23.1 exhibited different but closely related restriction endonuclease patterns with common fragments present in both phages (Fig. 2A).

#### RAPD-PCR analysis

RAPD-PCR analyses were run in triplicate to assure consistent results. No amplification products were observed in reactions performed in the absence of DNA template. The RAPD profile of DNA isolated from *D*. *solani* IPO2222 was different that from ϕPD10.3 and ϕPD23.1. The ϕPD10.3 and ϕPD23.1 yielded different but closely related RAPD patterns (Fig. 2B).

#### PFGE analysis

**Fig 3:**
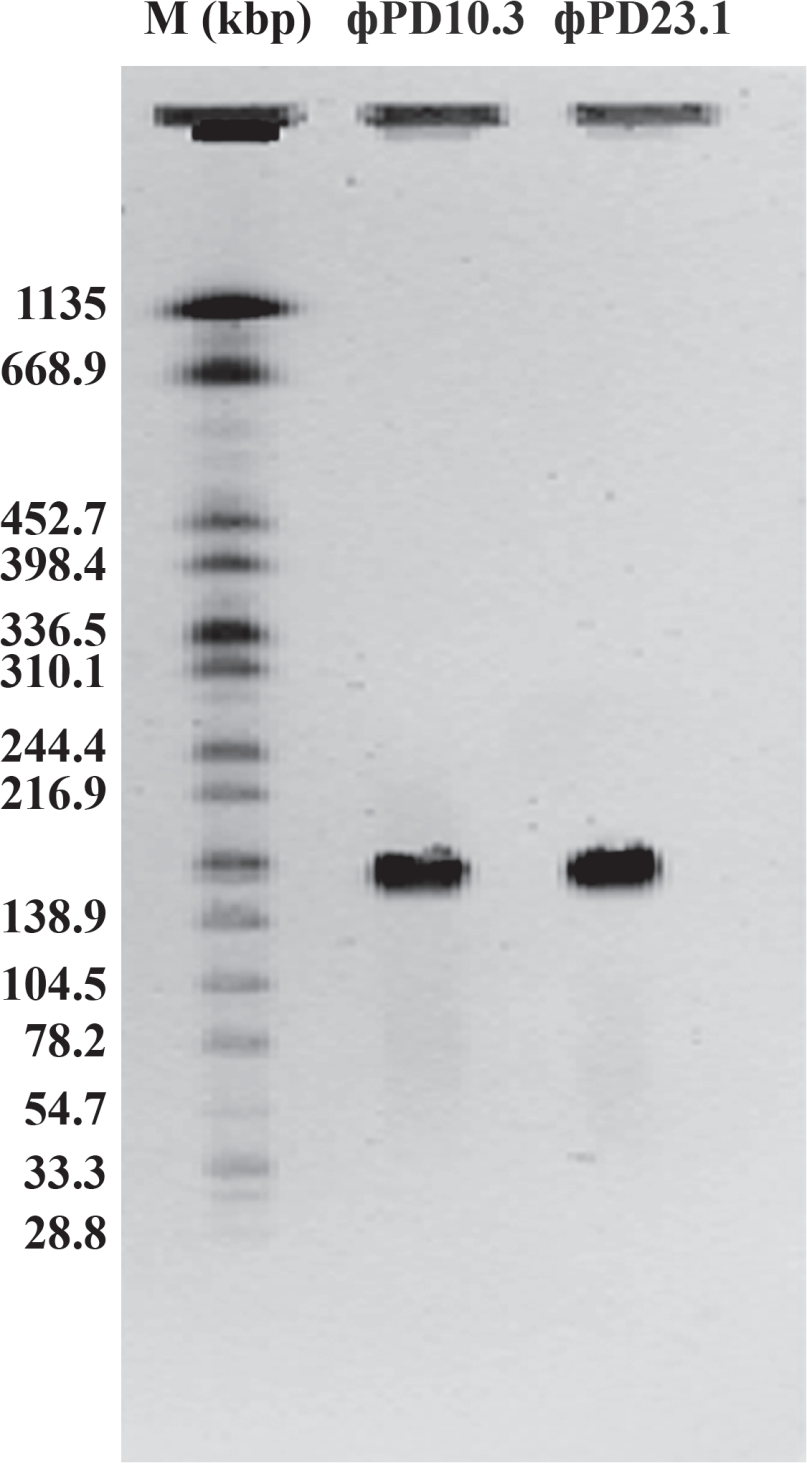
Estimation of bacteriophage ϕPD10.3 and ϕPD23.1 genome size by pulse-field gel electrophoresis (PFGE). Indigested with restriction endonucleases genomic DNA from phage ϕPD10.3 and ϕPD23.1 particles was run on 1% SeaKem Gold agarose in 0.5 x TBE buffer with 100 mM thiourea for 22 h at 14°C with the pulse time linearly increased from 2.2 to 54.2 s and a fixed reorientation angle of 120°. The gels were stained with 5 mg ml^-1^ ethidium bromide (Sigma), washed with water for 10 min and analyzed under UV light in GelDoc imaging system. As a marker the *Salmonella enterica* serotype Braenderup (strain H9812, ATCC) was used.

The PFGE analysis was performed on undigested genomic DNA with restriction endonucleases to estimate genome sizes of the ϕPD10.3 and ϕPD23.1. Both phages had genomes of similar size, *ca*. 180,000 to 190,000 bp (Fig. 3).

### Description of the ϕPD10.3 and ϕPD23.1 draft genomes and comparative genomic analysis

Draft genome sequences of ϕPD10.3 and ϕPD23.1 were obtained using Illumina next generation sequencing technology with the average of 100-fold coverage and assembled *de novo* at BaseClear, the Netherlands (www.baseclear.com). The reads were assembled into 28 (ϕPD10.3) and 29 contigs (ϕPD23.1) with a total length of 192, 291 bp (ϕPD10.3) and 188,540 bp (ϕPD23.1). For each phage the contigs were concatenated into pseudomolecules with spacers between contigs that introduced start and stop codons in all six possible reading frames to assure proper translation from all 6 possible ORFs at a single locus. The draft genome sequences (contigs) were deposited at DDBJ/EMBL/GenBank under accession numbers: KM209229—KM209273 (ϕPD10.3) and KM209274—KM209320 (ϕPD23.1) (S1 Fig., S2 Table). The raw Illumina reads of ϕPD10.3 and ϕPD23.1 were subsampled and processed using Mira assembler in order to close their genomes. The largest scaffold obtained for ϕPD10.3 contained 156,113 bp. (S1 File), whereas the largest scaffold for ϕPD23.1 contained 153,365 bp. (S2 File). We were not able to close the genomes: 36,178 bp. (ϕPD10.3) and 35,175 bp. (ϕPD23.1) of the raw Illumina reads could not be scaffolded.

The general features of the ϕPD10.3 and ϕPD23.1 genomes are summarized in Table 3. A pairwise comparison of the nucleotide sequences of ϕPD10.3 and ϕPD23.1 revealed that they had considerable similarity suggesting a common origin. The common genome (core genome), estimated with EDGAR, consists of 207 PEGs (protein encoding genes), whereas only 19 and 16 PEGs are specific for phage ϕPD10.3 and ϕPD23.1, respectively. The majority of genes found in one but absent in the other phage were those coding for hypothetical proteins with unknown function. Genomes of ϕPD10.3 and ϕPD23.1 share significant homology with the genome of *Dickeya* spp. lytic bacteriophage ϕD5 isolated by us in the previous study [22]; 193 genes (ϕPD10.3) and 189 genes (ϕPD23.1) have homologs in the genome of ϕD5. Interestingly, the majority of ϕPD10.3 and ϕPD23.1 genes do not have homologs in of T4—one of the best characterized broad host range *Enterobacteriaceae* phage. Both phages share only two genes with T4, namely phage recombination protein and *regA*—translational repressor of early genes. The lifestyle of ϕPD10.3 and ϕPD23.1 predicted from PHACTS indicated that both may be lytic bacteriophages.

**Table 3:** General features of the bacteriophage ϕPD10.3 and ϕPD23.1 genomes.

Feature	Bacteriophage ϕPD10.3	Bacteriophage ϕPD23.1	Bacteriophage ϕD5(Czajkowski *et al*. 2014)
**Genome size (bp)**	192 291	188 540	155 346
% **GC**	48.6%	49.25%	49.7%
% **of coding regions in the genome**	79.25%	83.15%	89.9%
**Number of predicted ORFs (PEGs)**	226	223	196
% **of PEGs with assigned functions**	37.2%—with assigned functions	39.5%—with assigned functions	25.5%—with assigned functions
	21.7%—unclassified with no assigned category	22.4%—unclassified with no assigned category	21.4%—unclassified with no assigned category
	7.5%—have unknown functions	8%—have unknown functions	4.1%—have unknown functions
**Average gene length (bp)**	553–686	538–704	711
**Number of PEGs in functional groups**	2—nucleotide		2—nucleotide
	metabolism		metabolism
	2—energy metabolism	1—nucleotide	1—energy
	16—transport and	metabolism	metabolism
	binding	1—energy metabolism	7—transport and
	15—DNA metabolism	17—transport and	binding
	4—transcription	binding	9—DNA metabolism
	1—protein fate	15—DNA metabolism	3—transcription
	2—regulatory	5—transcription	1—regulatory
	functions	7—cell envelope	functions
	4—cell envelope	9—cellular processes	4—cell envelope
	8—cellular processes	4—mobile or	6—cellular processes
	4—mobile or	Extrachromosomal	4—mobile or
	extrachromosomal	elements	extrachromosomal
	elements		elements
	15—DNA metabolism		
**Transcription start codon (% of genes using this start codon)**	ATG (87.3%)	ATG (85.3%)	ATG (94.4%)
	GTG (10.4%)	GTG (13.6%)	GTG (4.1%)
	TTG (2.3%)	TTG (1.1%)	TTG (1.5%)
**tRNAs**	tRNA-Met	tRNA-Met	tRNA-Met
	tRNA-Glu	tRNA-Glu	

The genomes of ϕPD10.3 and ϕPD23.1 did not contain any genes coding for toxins and allergens as tested by VirulenceFinder 1.2 and VirulentPred. A search in BLAST did not reveal the presence of any possible toxins, allergens and antibiotic resistance genes in the genomes of either bacteriophage.

### Structural proteome of ϕPD10.3 and ϕPD23.1 bacteriophages

**Fig 4:**
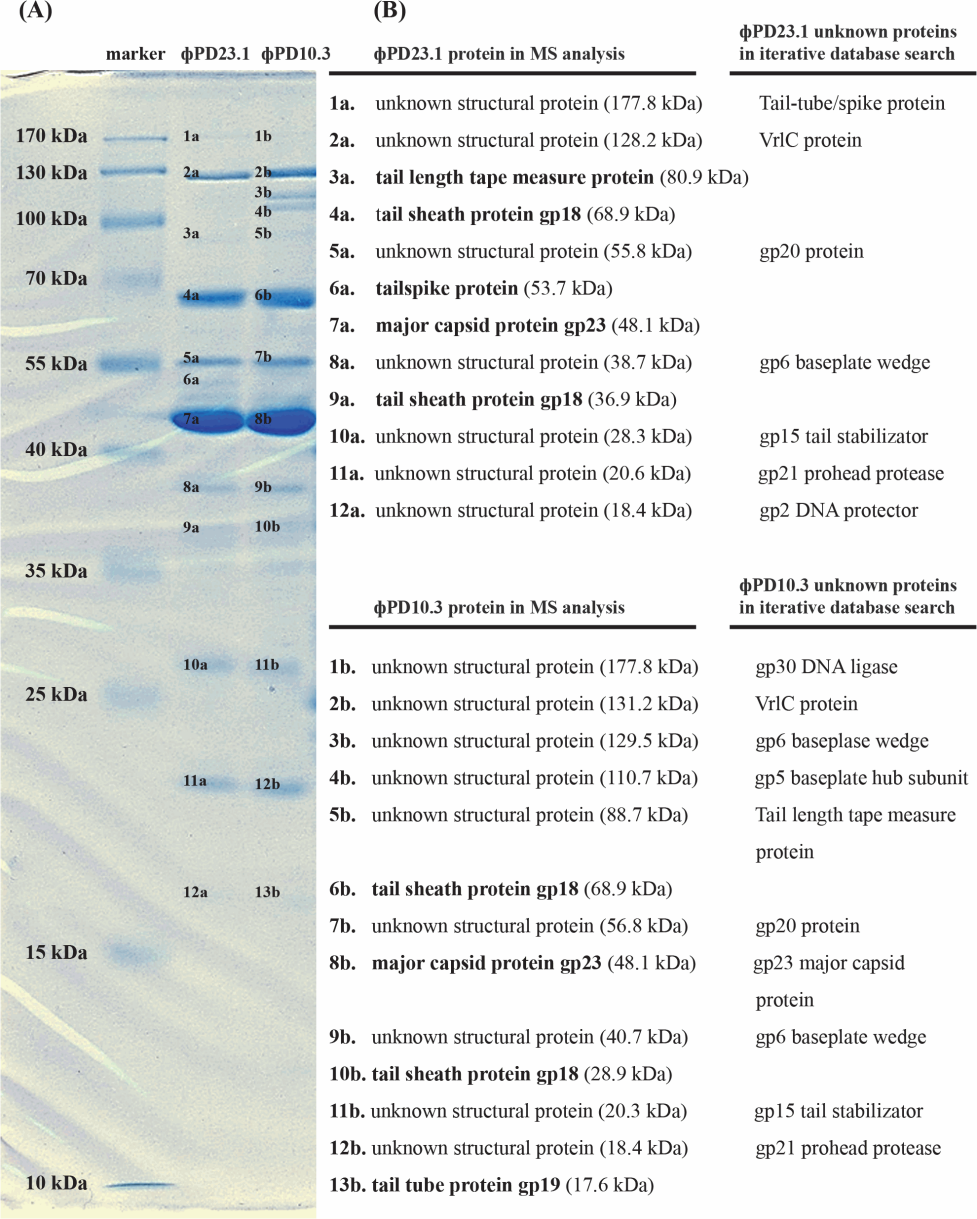
Bacteriophage ϕPD10.3 and ϕPD23.1 structural proteins separated in SDS-PAGE gels (A) and identification of bacteriophage ϕPD10.3 and ϕPD23.1 structural proteins with mass spectrometry (B). (A) For SDS-PAGE electrophoresis *ca*. 5 x 10^9^ pfu ml^-1^ were mixed with Laemmli buffer and frozen in liquid nitrogen for 1–2 min. following by boiling at 95°C for 5 min. The phage proteins were separated in 12% acrylamide SDS-PAGE gel for 19 h at 50 V at 22°C. PageRuler Pre-stained Protein Ladder (Thermo Scientific), prepared according to instructions provided by manufacturer, was used as a size marker. The gel was stained with PageBlue Coomasie Blue (Thermo Scientific) for 18 h and destained with sterile demineralized water for 6 h at room temperature (22°C) as suggested by the manufacturer. (B) For MS analysis of phage structural proteins, protein bands obtained for bacteriophage ϕPD10.3 and ϕPD23.1 were excised from gel with a sterile scalpel and sent for mass spectrometry analysis to Mass Spectrometry Laboratory, Institute of Biochemistry and Biophysics, Polish Academy of Sciences in Warsaw, Poland. Furthermore, possible molecular functions of the unknown structural proteins obtained from SDS-PAGE (A) phage ϕPD10.3 and ϕPD23.1 proteins against the sequence database of known three-dimensional (3D) protein structures was done using PSI-BLAST accessed *via* website of NCBI (http://www.ncbi.nlm.nih.gov/) [52] and using GeneSilico Protein Structure Prediction Meta-server (https://genesilico.pl/meta2/acl_users) [51]. The protein predictions with the highest scores were considered as the most valid [51, 52].

Electrospray ionization-MS/MS analysis of gel-separated ϕPD10.3 and ϕPD23.1 proteins led to the identified 13 and 12 phage proteins, respectively (Fig. 4). Of these, only have assigned functions based on sequence similarities with other phage proteins. The most abundant proteins in both samples were the major capsid protein (gp23) and tail sheath protein (gp18), as well as, in the ϕPD10.3 proteome tail tube protein (gp19) and the ϕPD23.1 proteome tail length tape measure protein and tailspike protein. Nine and 7 proteins present in ϕPD10.3 and ϕPD23.1 proteomes, respectively, were characterized as unknown structural proteins, to which function could not be assigned.

We were able to assign functions to all unknown structural proteins obtained in SDS-PAGE by comparison of protein 3D structure databases (Fig. 4). All the predicted proteins were phage-associated and functionally-involved in bacteriophage particle assembly.

### Suppression of soft rot development on potato slices and whole tubers co-inoculated with *P*. *carotovorum* subsp. *carotovorum* Ecc71, *D*. *solani* IPO2222, *P*. *wasabiae* SCC3193 and ϕPD10.3 and ϕPD23.1 bacteriophages

**Fig 5:**
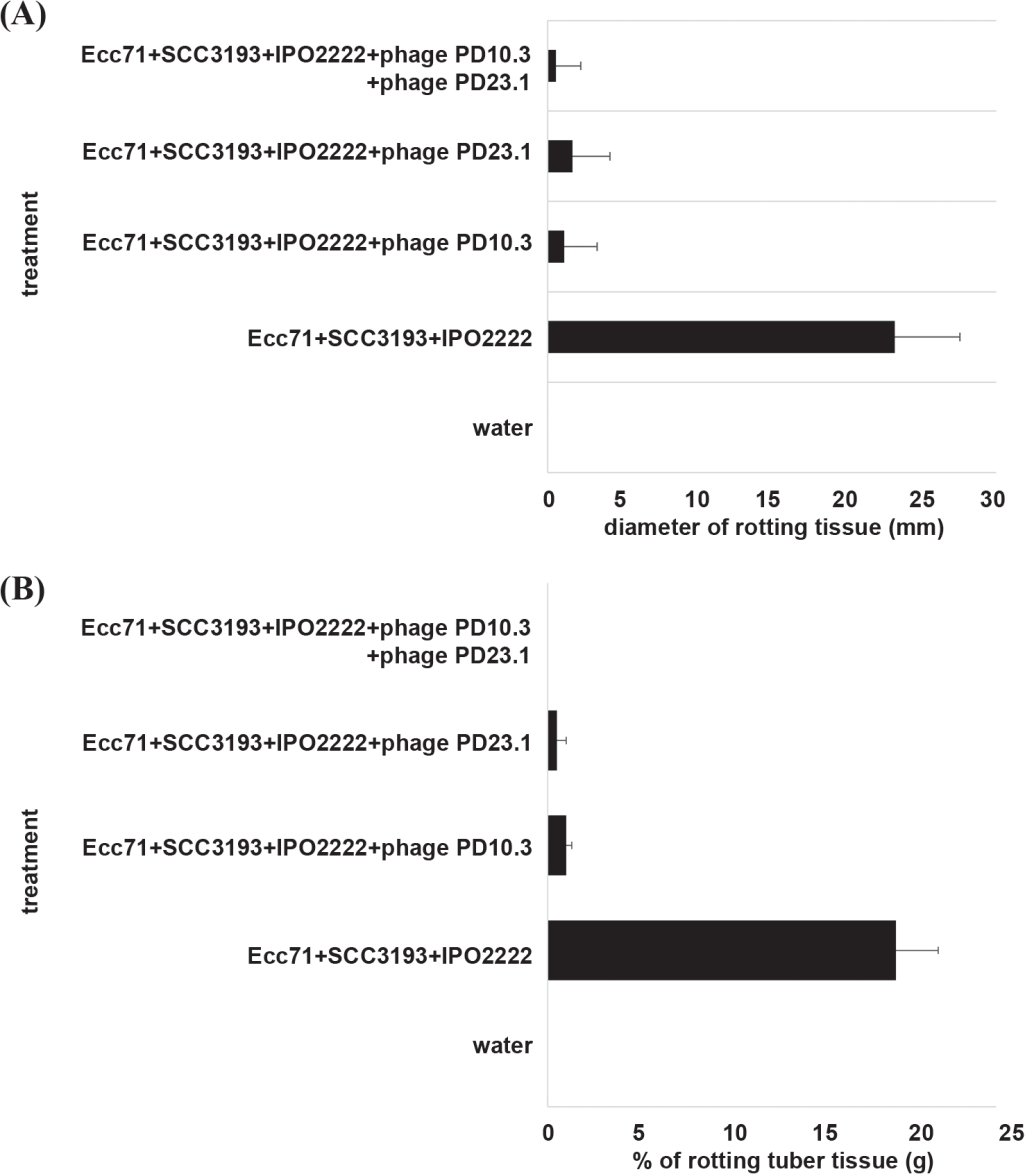
Protection effect on ϕPD10.3 and ϕPD23.1 on potato tuber slices (A) and whole tubers (B) against *D*. *solani* IPO2222, *P*. *carotovorum* subsp. *carotovorum* Ecc71 and *P*. *wasabiae* 3139. (A) Reduction of maceration ability of mixture of *D*. *solani* IPO2222, *P*. *carotovorum* subsp. *carotovorum* Ecc71 and *P*. *wasabiae* 3139 co-inoculated with bacteriophage ϕPD10.3 and ϕPD23.1 in potato tubers slices. The effect was determined by measuring the diameter of rotting tissue (in mm) after 72 h incubation at 28°C in a humid box. Wells of potato slices were filled up with 50 μl of sterile water (negative control), with 50 μl of bacterial suspension in water containing 10^7^ cfu ml^-1^ of *D*. *solani* IPO2222, *P*. *carotovorum* subsp. *carotovorum* Ecc71 and *P*. *wasabiae* 3139 (positive control) or with 50 μl of bacterial suspension in water containing 10^7^ cfu ml^-1^ of *D*. *solani* IPO2222, *P*. *carotovorum* subsp. *carotovorum* Ecc71 and *P*. *wasabiae* 3139 and 10^5^ pfu ml^-1^ of ϕPD10.3 and/or ϕPD23.1 bacteriophage. Three potato slices containing 3 wells each and derived from three different tubers were used per experiment. The experiment was independently repeated one time and the results were averaged. (B) Reduction of infection ability of mixture of *D*. *solani* IPO2222, *P*. *carotovorum* subsp. *carotovorum* Ecc71 and *P*. *wasabiae* 3139 co-inoculated with bacteriophage ϕPD10.3 and ϕPD23.1 in the vascular tissue of potato tubers. The effect was determined by measuring the percentage of rotting tuber tissue (in g) after incubation the tubers at 28°C in a humid box. Caps of rose ends were removed from tubers with a sterile knife and resulting capless tubers were inoculated with 100 μl of bacterial suspension in water containing 10^7^ cfu ml^-1^ of *D*. *solani* IPO2222, *P*. *carotovorum* subsp. *carotovorum* Ecc71 and *P*. *wasabiae* 3139 (positive control) or with 100 μl of bacterial suspension in water containing 10^7^ cfu ml^-1^ of *D*. *solani* IPO2222, *P*. *carotovorum* subsp. *carotovorum* Ecc71 and *P*. *wasabiae* 3139 and co-inoculated with 100 μl of 10^5^ pfu ml^-1^ of ϕPD10.3 and/or ϕPD23.1 bacteriophage. Ten potato tubers were used per treatment. The experiment was independently repeated one time and the results were averaged.

The ϕPD10.3 and ϕPD23.1 were tested in a potato slice assay and whole tuber assay for their ability to reduce or prevent maceration of potato tuber tissue by *P*. *carotovorum* subsp. *carotovorum* Ecc71, *D*. *solani* IPO2222 or *P*. *wasabiae* SCC3193. We tested the bacteriophages in a worst case scenario in which we simulated infection with multiple soft rot *Enterobacteriaceae* pathogens. In replicated *in vitro* experiments, the two bacteriophages individually or co-inoculated with a combination of bacteria (Ecc71+IPO2222+SCC3193), were able to significantly reduce potato tuber tissue maceration by at least 80% of the control potato slices inoculated with a mixture of bacteria only and by at least 95% of the control whole tubers inoculated with a mixture of bacteria (Fig. 5)

## Discussion

This study assessed the presence of lytic bacteriophages infecting blackleg and soft rot causing *Enterobacteriaceae* currently prevailing in potato in Europe *viz*. *D*. *solani*, *P*. *carotovorum* subsp. *carotovorum* and *P*. *wasabiae*. We focused on broad host range bacteriophages able to infect three SRE species most commonly associated with potato diseases in Europe. To our knowledge, this is the first study in which lytic bacteriophages able to infect more than one SRE species have been characterized in detail.

The frequency of isolation of lytic bacteriophages able to lyse the SRE bacteria in our study was relatively low, but was comparable to that in previous studies [21, 22, 53]. For example, lytic bacteriophages against *Dickeya* spp. were isolated from only 22% of soil samples [22] and Gross *et al*. (1991) reported that only 5% of potato rhizosphere soil samples contained bacteriophages against *P*. *carotovorum* subsp. *carotovorum* [53]. Of the 28 phages isolated in this study, 32.2% were isolated from (symptomatic) potato tubers (9 phages), 28.6% from bulk soil (8 phages), 25% from potato rhizosphere soil (7 phages) and 14.2% from (symptomatic) potato stems (4 phages) (Table 1). There is no information available in the literature addressing whether bacteriophages infecting *Pectobacterium* and *Dickeya* species are more frequently isolated from plants and soils infested with SRE than when the pathogens are absent. It can be speculated that the presence of viable host bacteria would favor the occurrence of bacteriophages. However, no correlation between the presence of SRE bacteria and bacteriophages was found in plant samples in this study (data not shown). As found previously (22), for all 164 samples tested here, an enrichment of phage particles in bacterial host cultures was required prior to phage isolation. However, the low recovery of bacteriophages from plant and soil samples this time cannot be explained by the seasonal differences. The phages were isolated during the spring/summer (April—September) when the highest populations of host bacteria can be expected, and from symptomatic plants harboring high bacterial populations [54].

All phages isolated in this study belonged to the order *Caudovirales* and family *Myoviridae* based on their morphology determined by transmission electron microscopy (Fig. 1). In addition, all of them have icosahedral heads and contractile tails of the diameter and sizes which would classify them as morphotype A1 [55]. Different studies have shown that more than 90% of lytic bacteriophages isolated and characterized with transmission electron microscopy belong to the order *Caudovirales* [55–57]. Previously [22] we reported the isolation of nine bacteriophages infecting *Dickeya* spp., belonging to the *Myoviridae* family [22]. Likewise, Adriaenssens and co-workers (2012) and Ravensdale *et al*. (2007) also isolated and characterized *Myoviridae* bacteriophages against *D*. *solani* and *P*. *carotovorum*, respectively [23, 24], suggesting that the SRE-infecting bacteriophages belonging to the family *Myoviridae* are the most abundant in the environment.

The large majority of phages (93%) were able to infect isolates of both *P*. *carotovorum* subsp. *carotovorum* and *D*. *solani* but only ϕPD10.3 and ϕPD23.1 (7% of isolated bacteriophages) were able to infect *D*. *solani*, *P*. *carotovorum* subsp. *carotovorum* and *P*. *wasabiae* strains as well. None of the bacteriophages was able to infect either *P*. *atrosepticum* or *P*. *carotovorum* subsp. *brasiliense* isolates. This may reflect recent changes in population dynamics of the host bacteria in the environment in Europe. In the past, *P*. *atrosepticum* was the main potato pathogen in Europe responsible for blackleg disease [58] and it is still the case in Scotland, however, since 2005 the incidence of disease caused by the pathogen has decreased significantly [29, 59]. There are also no reports of the occurrence of *P*. *carotovorum* subsp. *brasiliense* in Poland and an only limited number of reports describes the presence of *P*. *carotovorum* subsp. *brasiliense* in other European countries [60]. In contrast, since 2005, *D*. *solani* has become well established in the potato ecosystem in Europe [6] and that *P*. *carotovorum* subsp. *carotovorum* and *P*. *wasabiae* are known to be present in Europe for a long time in association with potato plants [1, 13, 61].

ϕPD10.3 and ϕPD23.1 were characterized in detail. They were indistinguishable from each other based on their morphology, their host range on 99 isolates of *Pectobacterium* and *Dickeya* species and their genome size estimated using PFGE. Restriction fragment length polymorphism (RFLP) analysis and randomly amplified polymorphic DNA fingerprinting (RAPD) showed that the ϕPD10.3 and ϕPD23.1 were genetically very similar although differences in RFLP and RAPD profiles could be observed. Comparison of the ϕPD10.3 and ϕPD23.1 genomes showed high homology in organization and composition of their genomes. Ninety two percent of ORFs in both bacteriophages were homologous as evidenced by comparative analysis using EDGAR. ϕPD10.3 and ϕPD23.1 bacteriophages also showed reasonably high similarity with the previously described *Dickeya* spp. bacteriophage LIMEstone1 [23] and ϕD5 [62]. Surprisingly, ϕPD10.3 and ϕPD23.1 do not share significant gene homology with *Enterobacteriaceae* bacteriophage T4 as seen for other *Myoviridae* T4-related bacteriophages [63]. There is no straightforward explanation of this phenomenon especially when considering the fact that the *Myoviridae* bacteriophages described to date have in majority very similar genome organizations [63]. It may be that the ϕ10.3 and ϕ23.1 phages are members of a new, T4-related family of viruses; however, to prove this, additional studies will be required and are now being conducted. Unfortunately, we were unable to close the genomes of ϕPD10.3 and ϕPD23.1 probably due to in part a lack of similar phage genome sequences available in international databases for use as scaffolds. The ordering of scaffolds based on the recently described ϕD5 and LimeStone1 bacteriophages was suboptimal.

The great similarity of the ϕPD10.3 and ϕPD23.1 genomes may indicate a common origin for these phages; they were isolated however from potato tuber and stem samples, respectively, originating from two different regions in Poland (Table 1). The ϕPD10.3 and ϕPD23.1 proteomes were relatively highly similar to that of LimeStone1 and ϕD5. Currently, there is only limited number of publications describing structural phage proteomes and, to our knowledge, except the work of Adriaenssens *et al*. (2012), there are no reports describing proteomes of bacteriophages infecting soft rot *Enterobacteriaceae*. Consequently, the majority of ϕPD10.3 and ϕPD23.1 proteins could only be characterized as unknown structural proteins for which no matches were found in protein databases. To further explore the possible functions of these unknown phage proteins we used GeneSillico Protein Structure Prediction Meta-server and PSI-BLAST as advised by others [51, 52]. Based on the three-dimensional protein structures we could predict their functions but with different levels of confidence. These predictions need to be supported with experimental data.

One-step growth, adsorption studies and analysis of the phage genomes supported the use of ϕPD10.3 and ϕPD23.1 bacteriophages as biological control agents. Both phages infect a number of SRE strains belonging to different species and isolated from different environments and countries. They both showed rapid adsorption (70–83% adsorption at 20 min depending on the host) to bacterial cells and large burst size of 82 to 102 phages per infected cell (Table 2). Furthermore, the bioinformatic analysis of the ϕPD10.3 and ϕPD23.1 genomes indicated that they lack known genes coding for toxins, potential allergens, transposases or integrases (S1 Fig. and S2 Table). The lifestyle of ϕPD10.3 and ϕPD23.1 predicted with PHACTS indicated that both phages are lytic and not lysogenic. Moreover, we did not find any bacterial sequences in the genome nor any bacterial proteins in the phage proteomic analysis, and thus no generalized transduction of the bacterial hosts was not expected to occur. Bacteriophages are generally recognized as safe in the food industry and in veterinary medicine, and accordingly, products contains *Myoviridae* bacteriophages against *Listeria monocytogenes*, *Campylobacter jejuni*, *Staphylococcus aureus* are in use and are commercially available (for review see [64]).

In potato slice and whole tuber assays ϕPD10.3 and ϕPD23.1 were able to protect potato tuber tissue from maceration caused by a mixture of *P*. *carotovorum* subsp. *carotovorum*, *P*. *wasabiae* and *D*. *solani*. These bacteriophages were tested in the worst case scenario, in which bacterial isolates of different species were present in potato tuber tissue at relatively high numbers (5×10^5^ cfu per inoculation) (10^7^ cfu ml^-1^) as it may occur during natural infections. Protection was conferred under warm (28°C) and humid (80% relative humidity) conditions favorable for development of soft rot on inoculated potato slices. The bacteriophages both individually and when applied together were able to reproducibly and significantly reduce soft rot infections by at least 80% to 95% in comparison to controls inoculated with a mixture of bacteria only. In the host range experiments, both ϕPD10.3 and ϕPD23.1 bacteriophages were able to infect a range of different strains of *P*. *carotovorum* subsp. *carotovorum*, *P*. *wasabiae* and *D*. *solani*. These results suggest that these phages would be valuable biological control agents under natural field conditions, where high variation in host bacterial strains would be expected. *P*. *carotovorum* subsp. *carotovorum* strains isolated from potato are genetically diverse [1, 65], whereas the majority of reports describe *P*. *wasabiae* and *D*. *solani* as more genetically homogenous [6, 66]. While *P*. *wasabiae* and *D*. *solani* are recognized in Europe as dominant blackleg pathogens in the field, *P*. *carotovorum* subsp. *carotovorum* is the main cause of soft rot of potato in storage and transit [7]. ϕPD10.3 and ϕPD23.1 being able to infect all these species might be used in all stages of potato production from planting in the field to harvest and tuber storage.

One of the main difficulties in applying bacteriophages to control bacterial phytopathogens in agriculture is their high specificity, infecting only the particular strains of the target bacterial species [20]. This problem has been partially overcome in food industry [67] and in veterinary medicine [68] by using bacteriophage cocktails consisting of several phages differing in host specificity to maximize the host range covered. In the case of soft rot *Enterobacteriaceae* there are, however, no well-characterized and readily available bacteriophage collections from which phages with desired host specificities might be selected, prepared and used in biological control under field conditions. To our knowledge, there is also little information on the use of lytic bacteriophages against the *Pectobacterium* and *Dickeya* species, e.g. there is less than twenty research publications worldwide describing lytic bacteriophages against *Pectobacterium* and *Dickeya* species. Additionally, all lytic bacteriophages against soft rot *Enterobacteriaceae* described to date have a restricted host range and have not been tested against a large panel of bacterial strains. In addition, for the majority of bacteriophages infecting *Pectobacterium* and *Dickeya* species there is no information about their genome organization, nor on their stability under a variety of environmental conditions. We postulate that use of broad host range lytic bacteriophages infecting the SRE dominant in potato in Europe may help in minimizing losses caused by these pathogens during potato production. In principle, broad host lytic bacteriophages may be further combined with other approaches to control blackleg and soft rot infections of potato in an integrated strategy [8].

To fully explore the practical potential of broad host lytic bacteriophages against the dominant soft rot *Enterobacteriaceae* in potato in Europe, additional studies are needed such as the long-term efficiency and consistency of control in the field, including bacteriophage population survival and dynamics in bulk soil, in potato rhizosphere soil, in and on potato plants and tubers, application method, timing of application, formulation of bacteriophages in order to prolong their stability under harsh conditions and ecotoxicological risks. Greenhouse studies are now being conducted to directly assess the biological control properties of bacteriophages ϕPD10.3 and ϕPD23.1 against *P*. *carotovorum* subsp. *carotovorum*, *P*. *wasabiae* and *D*. *solani* infections in potato.

## Supporting Information

S1 Fig The draft genome sequence of bacteriophage ϕPD10.3 (A) and ϕPD23.1 (B). (PDF)

S1 Table *Pectobacterium* spp. and *Dickeya* spp. strains used in this study and the host range of 28 bacteriophages isolated initially against *D*. *solani* strain IPO222, *P*. *carotovorum* subsp. *carotovorum* strain Ecc71, *P*. *atrosepticum* strain SCRI 1043, *P*. *wasabiae* strain 3193, *P*. *carotovorum* subsp. *brasiliensis* strain LMG 21371 and *D*. *dianthicola* strain CFBP 1200. (DOCX)

S2 Table Summary of the 84 (ϕPD10.3) and 88 (ϕPD23.1.1) ORFs (PEGs) with predicted, assigned function. ORFs coding for hypothetical proteins and/or coding for conserved hypothetical proteins are not shown in the table.
(DOCX)

S1 File The largest scaffold (156,113 bp.) obtained for bacteriophage ϕPD10.3 using Mira assembler. (FASTA)

S2 File The largest scaffold (156,113 bp.) obtained for bacteriophage ϕPD23.1 using Mira assembler. (FASTA)
