# Supplementary material for: Genomic, Proteomic and Morphological Characterization of Two Novel Broad Host Lytic Bacteriophages ΦPD10.3 and ΦPD23.1 Infecting Pectinolytic Pectobacterium spp. and Dickeya spp
Source: PLoS One. 2015 Mar 24;10(3):e0119812. doi: 10.1371/journal.pone.0119812 (PMC4372400; doi:10.1371/journal.pone.0119812)
Supplement: S1 Fig — (PDF) [file pone.0119812.s001.pdf]

S1 Figure

A) The draft genome of bacteriophage ϕPD10.3

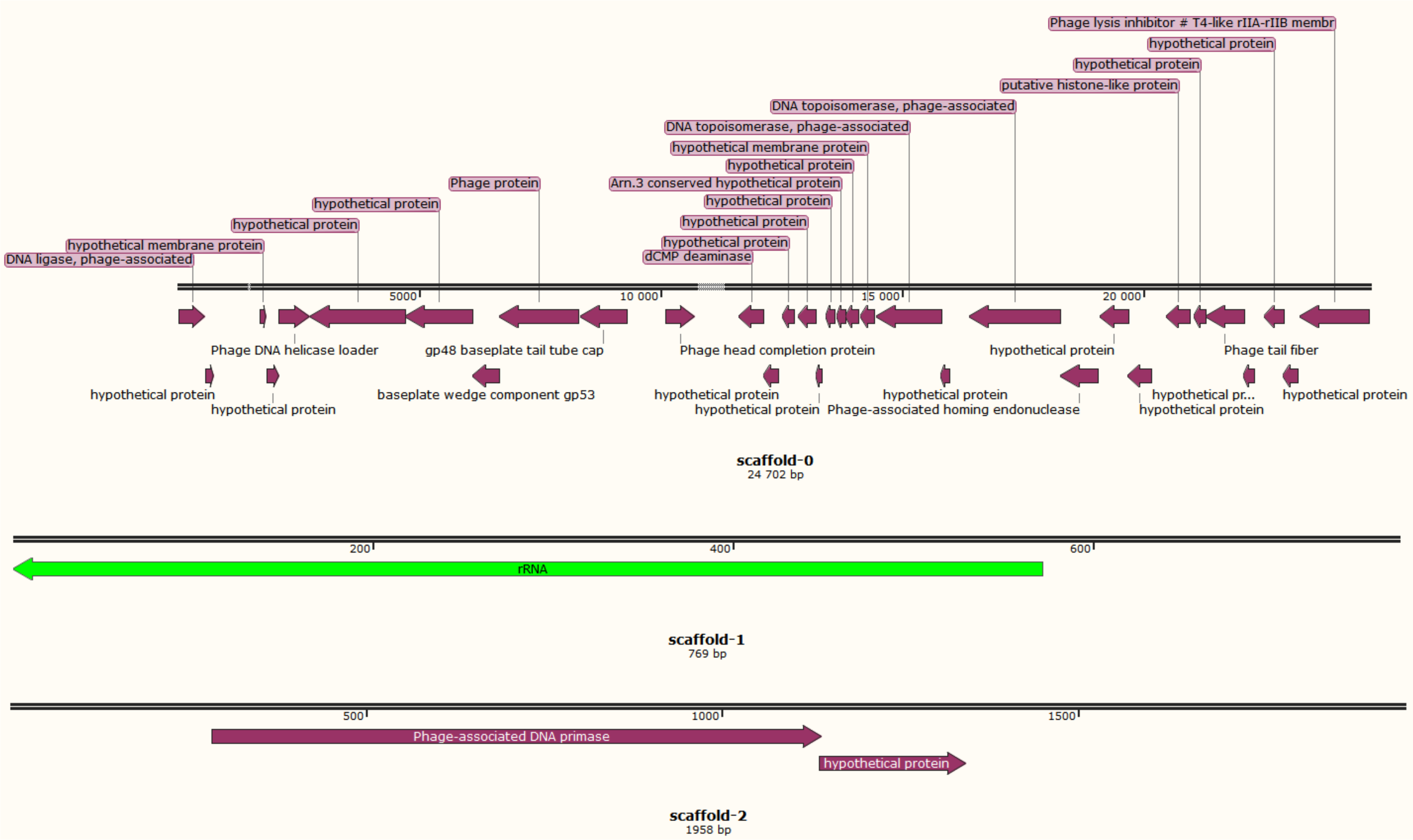

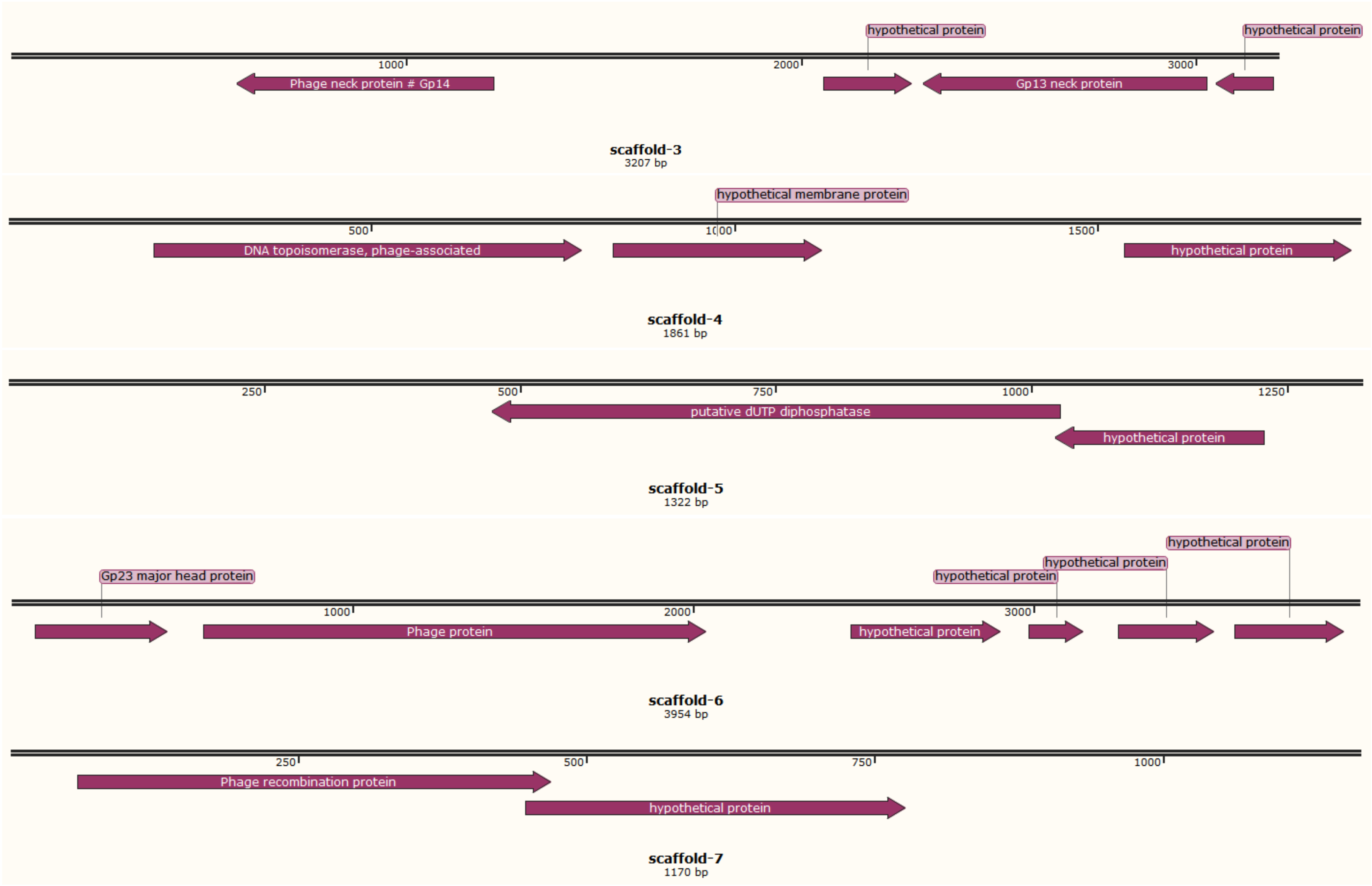
hypothetical protein
hypothetical protein
1000
2000
3000
Phage neck protein # Gp14
Gp13 neck protein
scaffold-3
3207 bp
hypothetical membrane protein
500
1000
1500
DNA topoisomerase, phage-associated
hypothetical protein
scaffold-4
1861 bp
250
500
750
1000
1250
putative dUTP diphosphatase
hypothetical protein
scaffold-5
1322 bp
hypothetical protein
hypothetical protein
Gp23 major head protein
hypothetical protein
1000
2000
3000
Phage protein
hypothetical protein
scaffold-6
3954 bp
250
500
750
1000
Phage recombination protein
hypothetical protein
scaffold-7
1170 bp

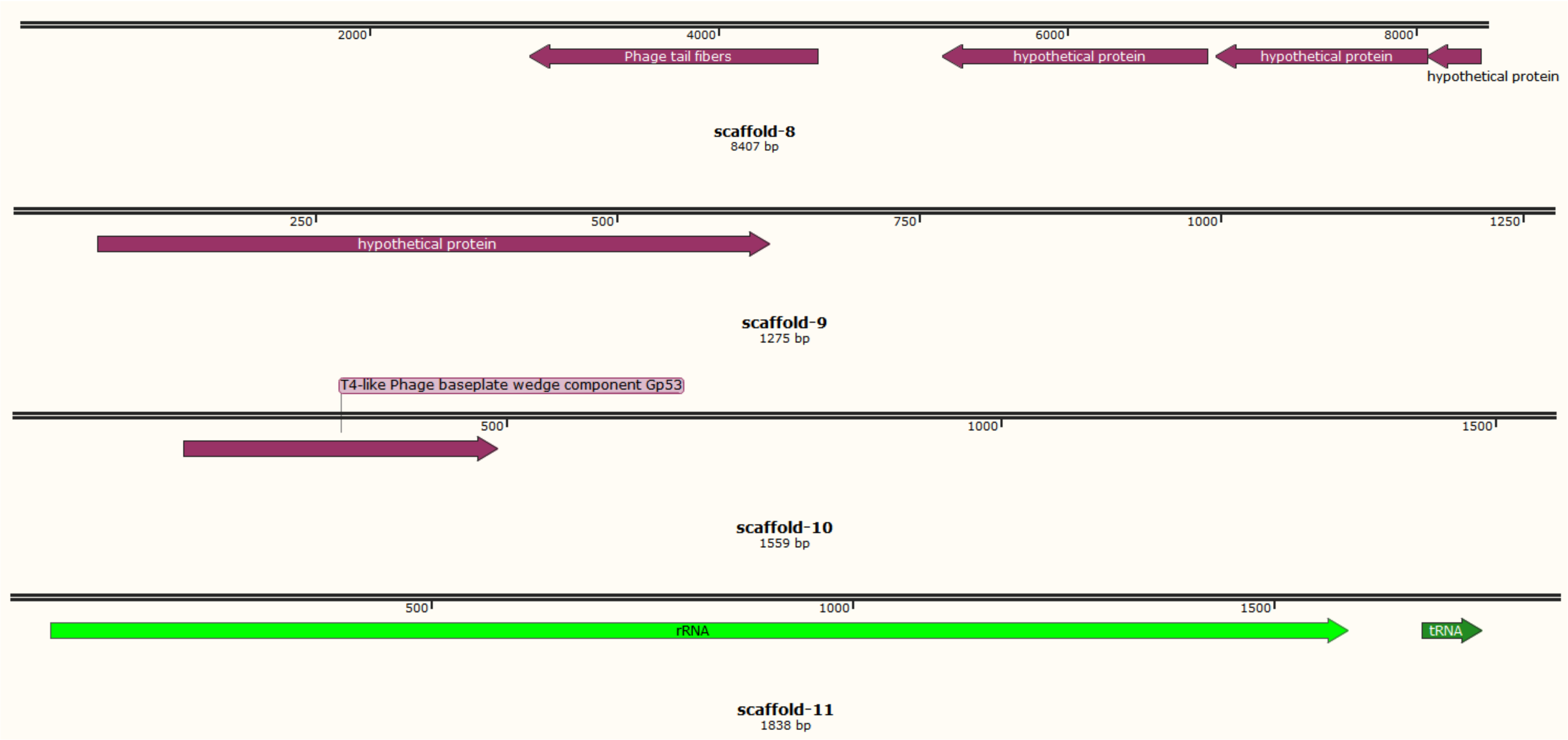
2000
4000
6000
8000
Phage tail fibers
hypothetical protein
hypothetical protein
hypothetical protein
scaffold-8
8407 bp
250
500
750
1000
1250
hypothetical protein
scaffold-9
1275 bp
T4-like Phage baseplate wedge component Gp53
500
1000
1500
scaffold-10
1559 bp
500
1000
1500
rRNA
tRNA
scaffold-11
1838 bp

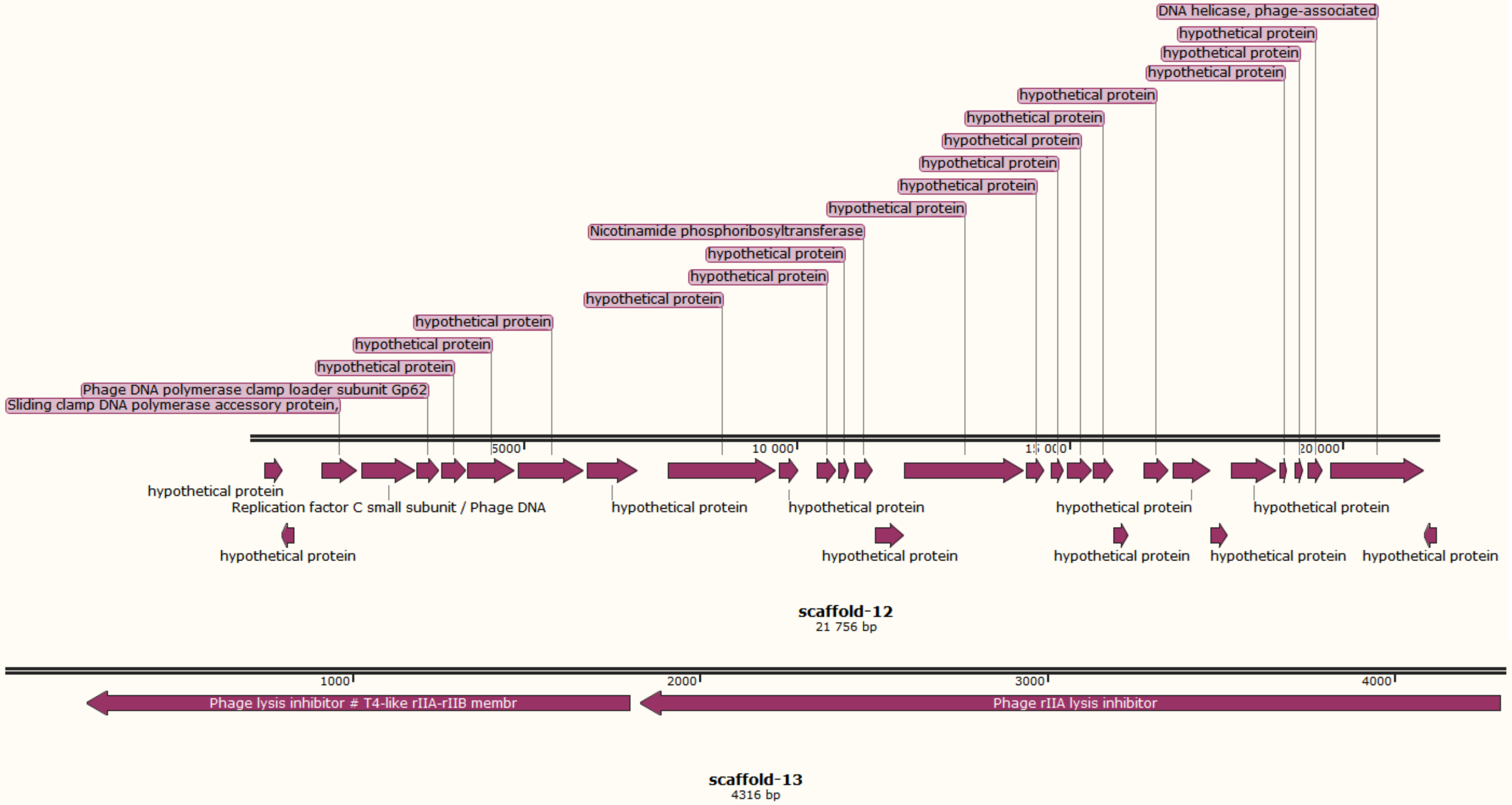
DNA helicase, phage-associated
hypothetical protein
hypothetical protein
hypothetical protein
hypothetical protein
hypothetical protein
hypothetical protein
hypothetical protein
hypothetical protein
hypothetical protein
Nicotinamide phosphoribosyltransferase
hypothetical protein
hypothetical protein
hypothetical protein
hypothetical protein
hypothetical protein
hypothetical protein
Phage DNA polymerase clamp loader subunit Gp62
Sliding clamp DNA polymerase accessory protein,
5000
10 000
15 000
20 000
hypothetical protein
Replication factor C small subunit / Phage DNA
hypothetical protein
hypothetical protein
hypothetical protein
hypothetical protein
hypothetical protein
hypothetical protein
hypothetical protein
hypothetical protein
hypothetical protein
scaffold-12
21 756 bp
1000
2000
3000
4000
Phage lysis inhibitor # T4-like rIIA-rIIB membr
Phage rIIA lysis inhibitor
scaffold-13
4316 bp

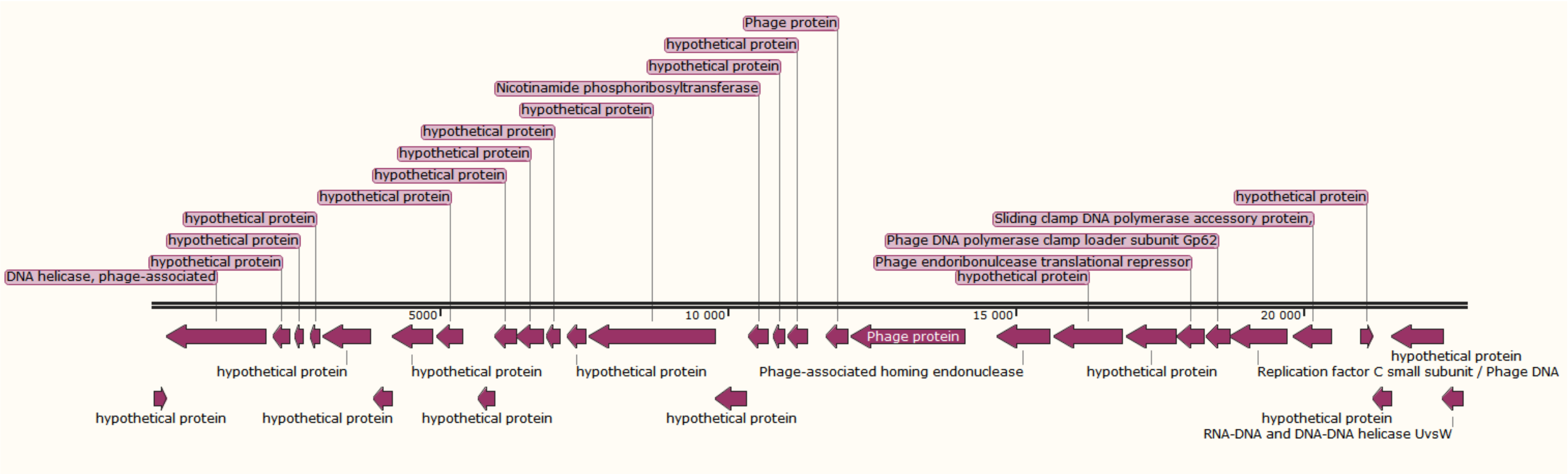

scaffold-14
22 815 bp

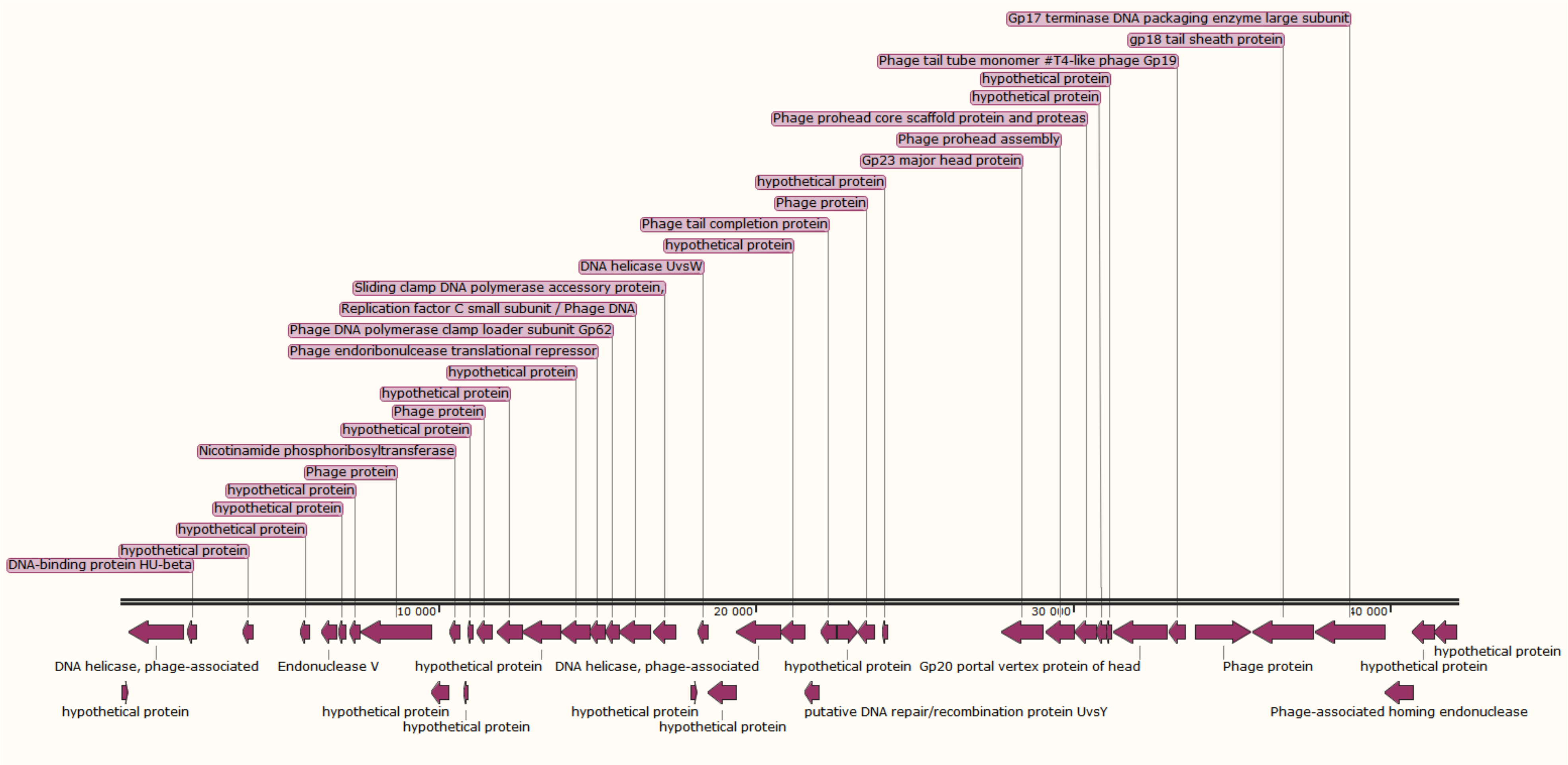

scaffold-15
42 113 bp

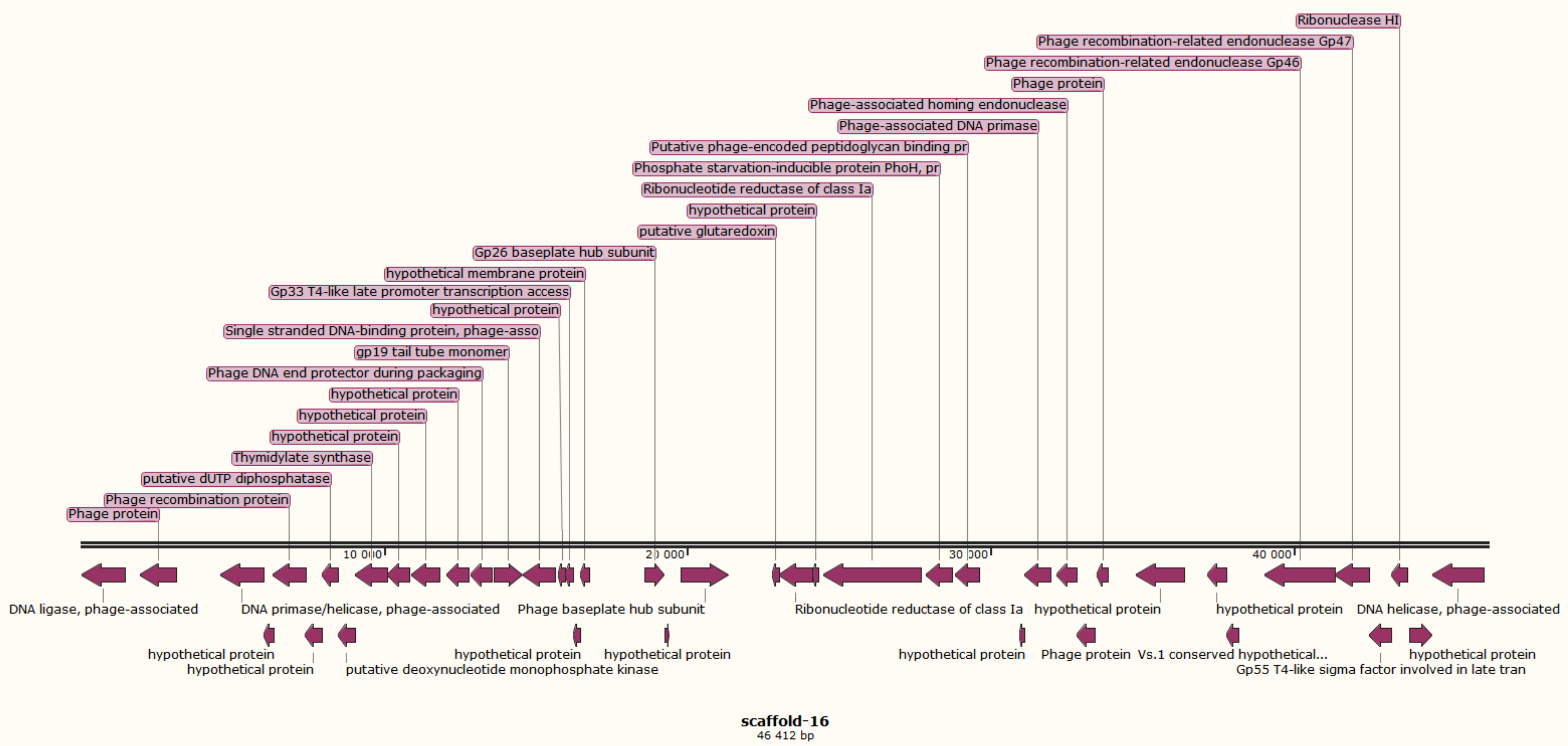
Ribonuclease HI
Phage recombination-related endonuclease Gp47
Phage recombination-related endonuclease Gp46
Phage protein
Phage-associated homing endonuclease
Phage-associated DNA primase
Putative phage-encoded peptidoglycan binding pr
Phosphate starvation-inducible protein PhoH, pr
Ribonucleotide reductase of class Ia
hypothetical protein
putative glutaredoxin
Gp26 baseplate hub subunit
hypothetical membrane protein
Gp33 T4-like late promoter transcription access
hypothetical protein
Single stranded DNA-binding protein, phage-asso
gp19 tail tube monomer
Phage DNA end protector during packaging
hypothetical protein
hypothetical protein
hypothetical protein
Thymidylate synthase
putative dUTP diphosphatase
Phage recombination protein
Phage protein
10 000
20 000
30 000
40 000
DNA ligase, phage-associated
DNA primase/helicase, phage-associated
Phage baseplate hub subunit
Ribonucleotide reductase of class Ia
hypothetical protein
hypothetical protein
DNA helicase, phage-associated
hypothetical protein
hypothetical protein
putative deoxynucleotide monophosphate kinase
hypothetical protein
hypothetical protein
hypothetical protein
Phage protein
Vs.1 conserved hypothetical...
Gp55 T4-like sigma factor involved in late tran
hypothetical protein
scaffold-16
46 412 bp

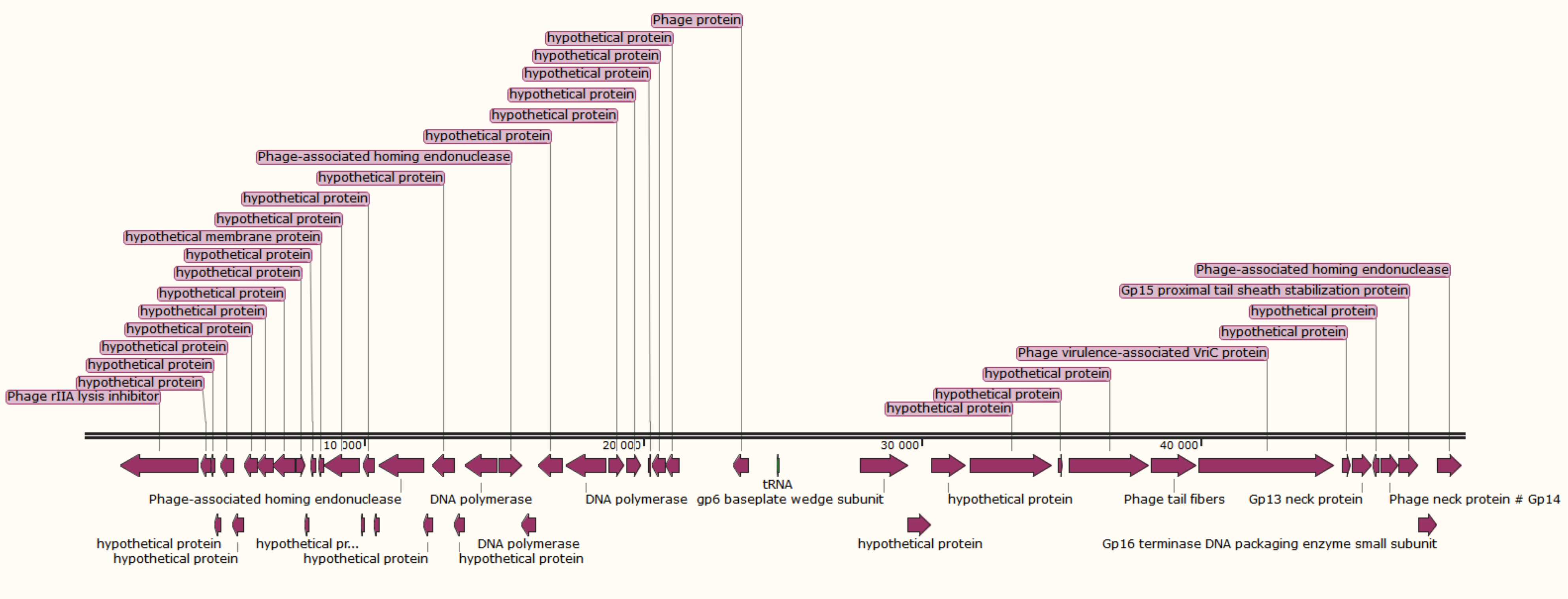

scaffold-17
49 430 bp

Supplementary Figure 1.

B) The draft genome of bacteriophage ϕPD23.1

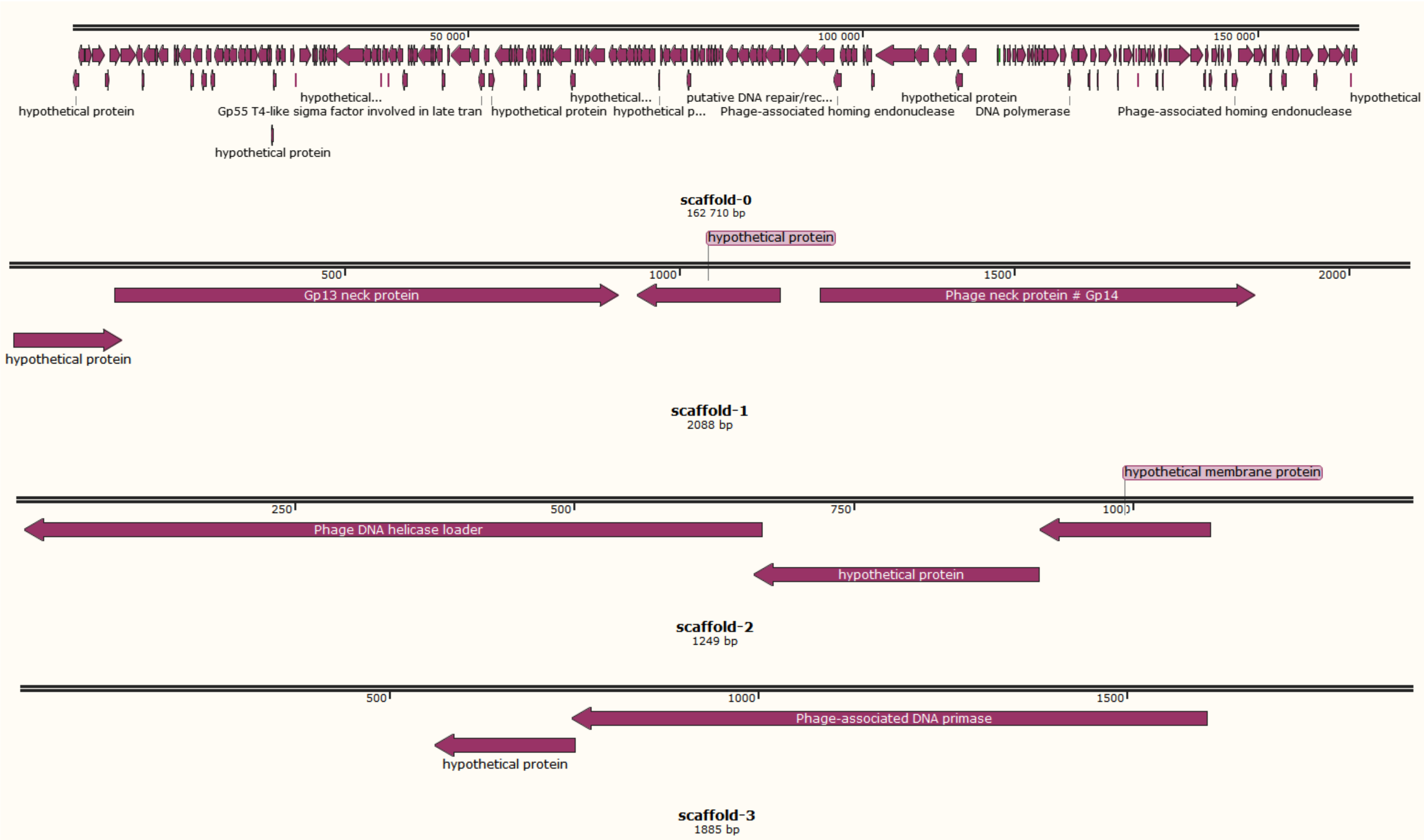

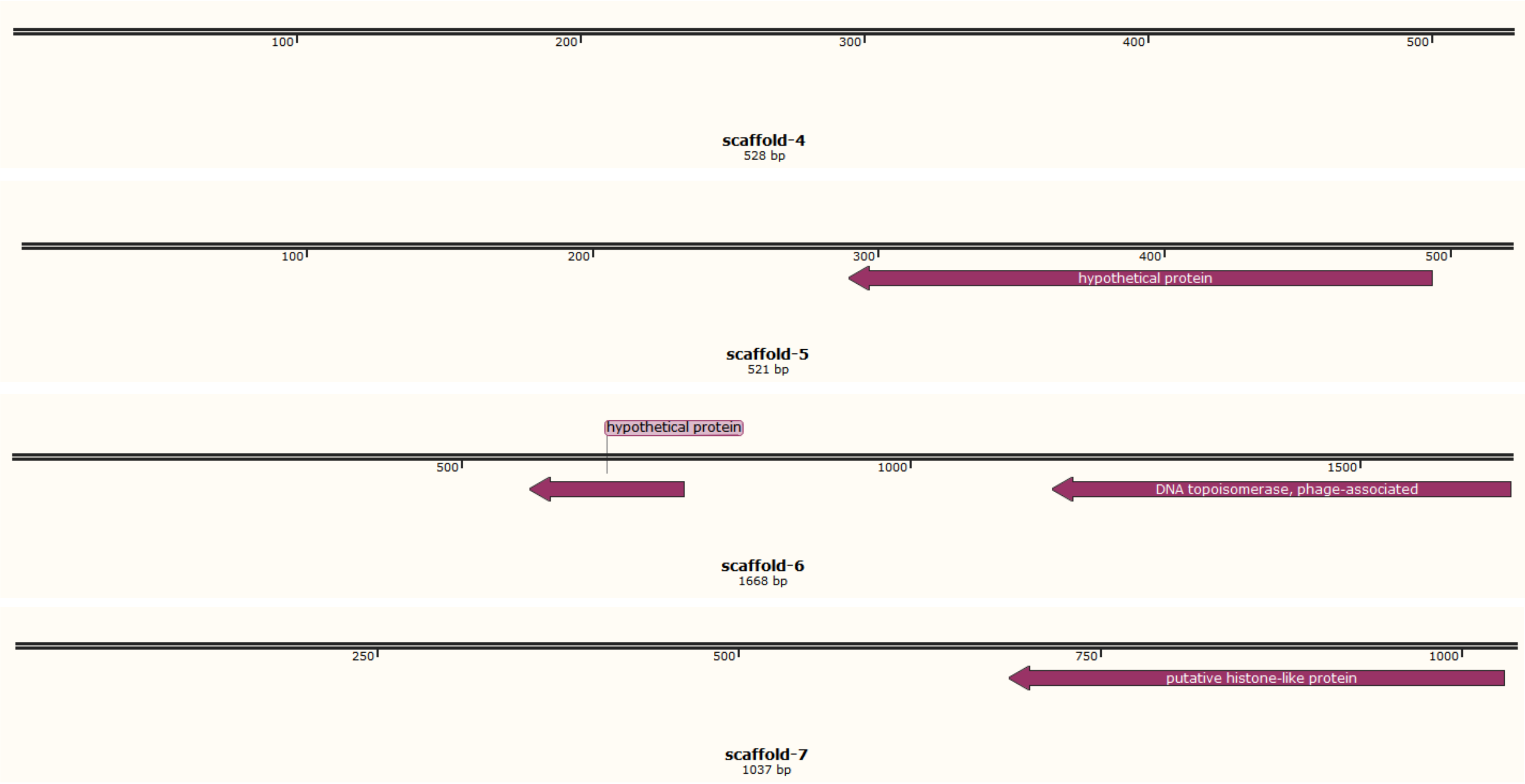

100
200
300
400
500
scaffold-4
528 bp
100
200
300
400
500
hypothetical protein
scaffold-5
521 bp
hypothetical protein
500
1000
1500
DNA topoisomerase, phage-associated
scaffold-6
1668 bp
250
500
750
1000
putative histone-like protein
scaffold-7
1037 bp

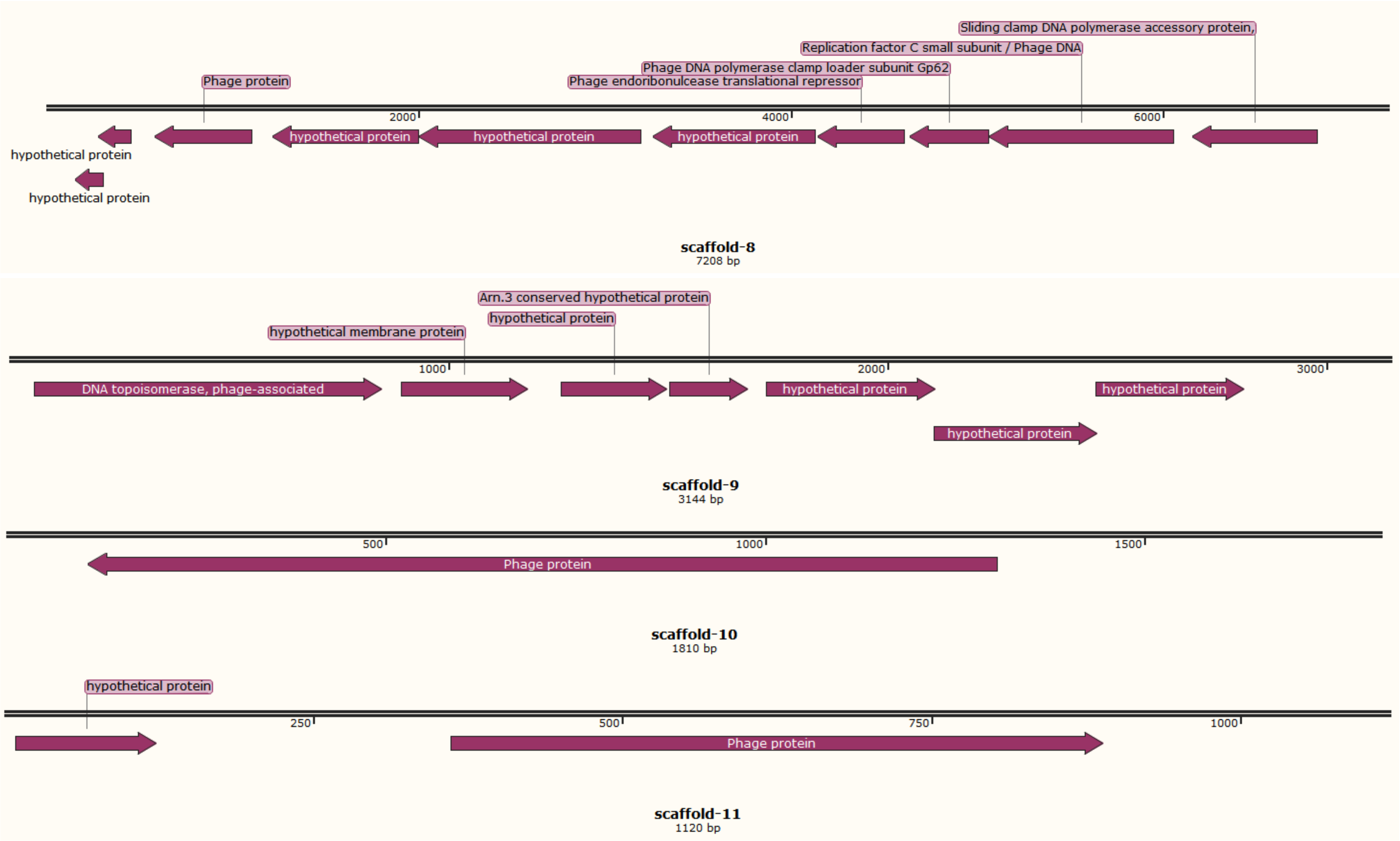
Sliding clamp DNA polymerase accessory protein,
Replication factor C small subunit / Phage DNA
Phage DNA polymerase clamp loader subunit Gp62
Phage endoribonulcease translational repressor
Phage protein
2000
4000
6000
hypothetical protein
hypothetical protein
hypothetical protein
hypothetical protein
hypothetical protein
scaffold-8
7208 bp
Arn.3 conserved hypothetical protein
hypothetical protein
hypothetical membrane protein
1000
2000
3000
DNA topoisomerase, phage-associated
hypothetical protein
hypothetical protein
hypothetical protein
scaffold-9
3144 bp
500
1000
1500
Phage protein
scaffold-10
1810 bp
hypothetical protein
250
500
750
1000
Phage protein
scaffold-11
1120 bp

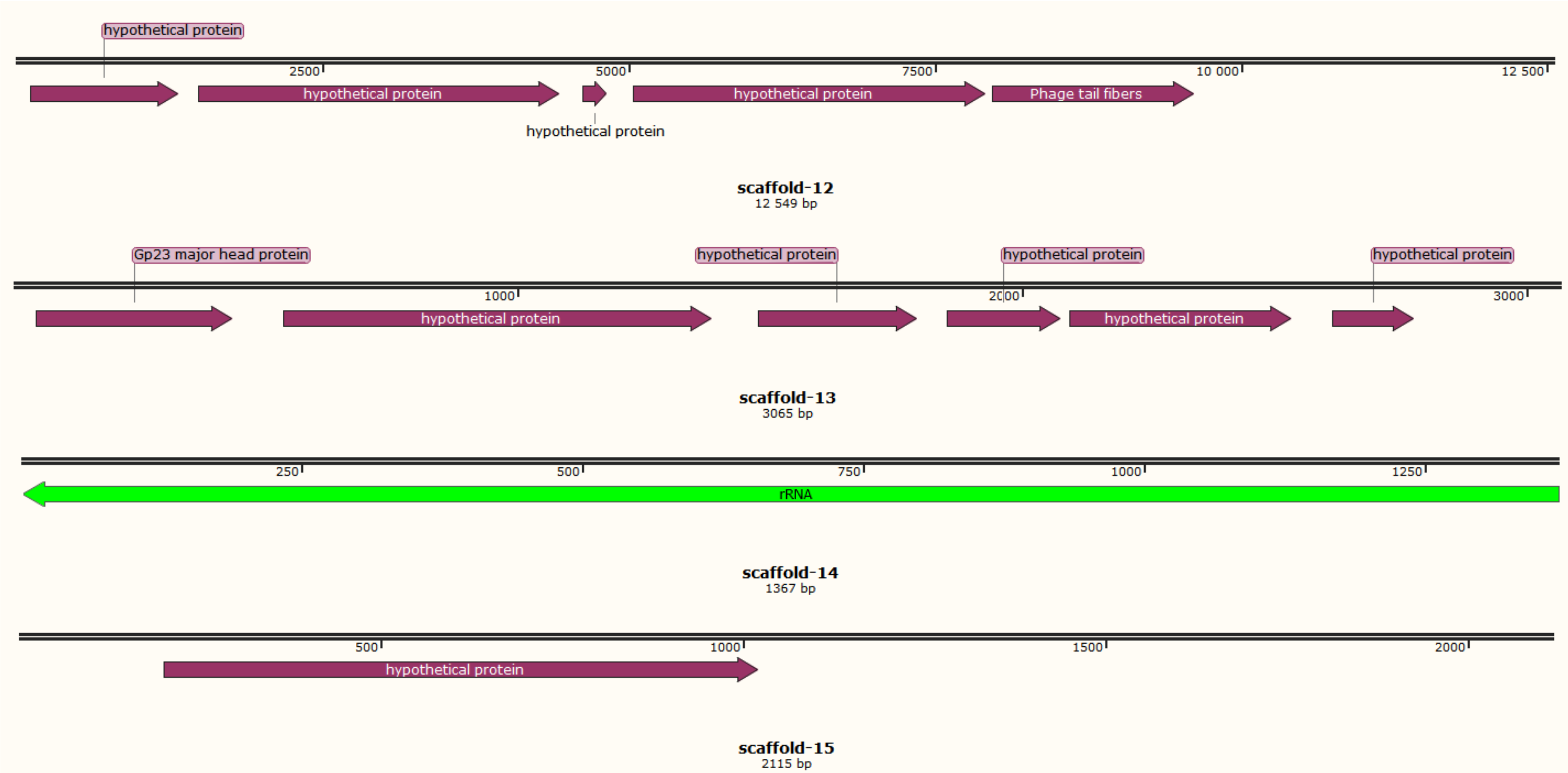
hypothetical protein
2500
5000
7500
10 000
12 500
hypothetical protein
hypothetical protein
Phage tail fibers
hypothetical protein
scaffold-12
12 549 bp
Gp23 major head protein
hypothetical protein
hypothetical protein
hypothetical protein
1000
2000
3000
hypothetical protein
hypothetical protein
scaffold-13
3065 bp
250
500
750
1000
1250
rRNA
scaffold-14
1367 bp
500
1000
1500
2000
hypothetical protein
scaffold-15
2115 bp
