# Supplementary material for: Genomic, Proteomic and Morphological Characterization of Two Novel Broad Host Lytic Bacteriophages ΦPD10.3 and ΦPD23.1 Infecting Pectinolytic Pectobacterium spp. and Dickeya spp
Source: PLoS One. 2015 Mar 24;10(3):e0119812. doi: 10.1371/journal.pone.0119812 (PMC4372400; doi:10.1371/journal.pone.0119812)
Supplement: S1 Table — (DOCX) [file pone.0119812.s002.docx]

**S2 Table.** *Pectobacterium* spp. and *Dickeya* spp. strains used in this study and the host range of 28 bacteriophages isolated initially against *D. solani* strain IPO222, *P. carotovorum* subsp. *carotovorum* strain Ecc71, *P. atrosepticum* strain SCRI 1043, *P. wasabiae* strain 3193, *P. carotovorum* subsp. *brasiliensis* strain LMG 21371 and *D. dianthicola* strain CFBP 1200.

| **Isolate** | **Species** | **Relevant characteristics (reference)** | **Other designation** | **Bacteriophages ^a^** | | | | | | | | | | | | | | | | | | | | | | | | | | | |
| --- | --- | --- | --- | --- | --- | --- | --- | --- | --- | --- | --- | --- | --- | --- | --- | --- | --- | --- | --- | --- | --- | --- | --- | --- | --- | --- | --- | --- | --- | --- | --- |
|  |  |  |  | **φ PD 2.1** | **φ PD 2.2** | **φ PD 3.1** | **φ PD 4.4** | **φ PD 4.6** | **φ PD 5.2** | **φ PD 5.4** | **φ PD 7.1** | **φ PD8.1** | **φ PD 8.5** | **φ PD 9.1** | **φ PD 10.3** | **φ PD 11.3** | **φ PD 11.4** | **φ PD 12.6** | **φ PD 15.3** | **φ PD 17.1** | **φ PD 18.2** | **φ PD 20.2** | **φ PD 22.1** | **φ PD 23.1** | **φ PD 25.1** | **φ PD 26.2** | **φ PD 27.2** | **φ PD 31.1** | **φ PD 31.2** | **φ PD 32.3** | **φ PD 33.3** |
| **3937** | ***D. dadantii*** | **Wildtype, *Saintpaula* sp., 1977** | - | - | - | - | - | - | - | - | - | - | - | - | - | - | - | - | - | - | - | - | - | - | - | - | - | - | - | - | - |
| **IFB 0010^T^** | ***D. dadantii*** | **Wildtype, *Pelargonium capitatum*, Comoro Islands, 1960** | **NCPPB 898, CFBP 1269** | - | - | - | - | - | - | - | - | - | - | - | - | - | - | - | - | - | - | - | - | - | - | - | - | - | - | - | - |
| **IFB 0023** | ***D. dadantii*** | **Wildtype, *Pelargonium capitatum*, Comoro Islands, 1960** | **SCRI 4069, B374** | - | - | - | - | - | - | - | - | - | - | - | - | - | - | - | - | - | - | - | - | - | - | - | - | - | - | - | - |
| **IFB 0064** | ***D. dadantii*** | **Wildtype, *Solanum tuberosum*, Peru** | **IPO598** | - | - | - | - | - | - | - | - | - | - | - | - | - | - | - | - | - | - | - | - | - | - | - | - | - | - | - | - |
| **IFB 0071** | ***D. dadantii*** | **Wildtype, *Solanum tuberosum*, Germany** | **IPO1260, 179** | - | - | - | - | - | - | - | - | - | - | - | - | - | - | - | - | - | - | - | - | - | - | - | - | - | - | - | - |
| **IFB 0068** | ***D. dadantii* subsp. *dieffenbachiae*** | **Wildtype, *Dieffenbachia* sp., the Netherlands** | **IPO1002** | - | - | - | - | - | - | - | - | - | - | - | - | - | - | - | - | - | - | - | - | - | - | - | - | - | - | - | - |
| **IFB 0025** | ***D. chrysanthemi* pv. *chrysanthemi*** | **Wildtype, *Chrysanthemum* sp., USA** | **1346, ICPB EC239** | - | - | - | - | - | - | - | - | - | - | - | - | - | - | - | - | - | - | - | - | - | - | - | - | - | - | - | - |
| **IFB 0050** | ***D. chrysanthemi* pv. *chrysanthemi*** | **Wildtype, *Chrysanthemum* sp., USA** | **ATCC11663, EC17** | - | - | - | - | - | - | - | - | - | - | - | - | - | - | - | - | - | - | - | - | - | - | - | - | - | - | - | - |
| **IFB 0055^T^** | ***D. chrysanthemi* pv. *chrysanthemi*** | **Wildtype, *Chrysanthemum morifolium*, USA, 1958** | **IPO2118, NCPPB 402, CFBP 2048** | - | - | - | - | - | - | - | - | - | - | - | - | - | - | - | - | - | - | - | - | - | - | - | - | - | - | - | - |
| **IFB 0017** | ***D. chrysanthemi* pv. *parthenii*** | **Wildtype, *Parthenium* sp., Denmark, 1957** | **1270** | - | - | - | - | - | - | - | - | - | - | - | - | - | - | - | - | - | - | - | - | - | - | - | - | - | - | - | - |
| **IFB 0069** | ***D. dadantii* subsp. *dieffenbachiae*** | **Wildtype *Solanum tuberosum*, Germany** | **IPO1259** | - | - | - | - | - | - | - | - | - | - | - | - | - | - | - | - | - | - | - | - | - | - | - | - | - | - | - | - |
| **IFB 0413** | ***D. dadantii* subsp. *dieffenbachiae*** | **Wildtype, China** | **JS4** | - | - | - | - | - | - | - | - | - | - | - | - | - | - | - | - | - | - | - | - | - | - | - | - | - | - | - | - |
| **IFB 0065** | ***D. dianthicola*** | **Wildtype, biovar 1, *Cichorium intybus*, the Netherlands** | **IPO1003** | - | - | - | - | - | - | - | - | - | - | - | - | - | - | - | - | - | - | - | - | - | - | - | - | - | - | - | - |
| **IFB 0074** | ***D. dianthicola*** | **Wildtype, biovar 7, *Solanum tuberosum*, the Netherlands** | **IPO996** | - | - | - | - | - | - | - | - | - | - | - | - | - | - | - | - | - | - | - | - | - | - | - | - | - | - | - | - |
| **IFB 0081** | ***D. dianthicola*** | **Wildtype, *Solanum tuberosum*, Spain** | **IPO1748, 1360-2236** | - | - | - | - | - | - | - | - | - | - | - | - | - | - | - | - | - | - | - | - | - | - | - | - | - | - | - | - |
| **IFB 0103^T^** | ***D. dianthicola*** | **Wildtype, *Dianthus caryophyllus*, UK, 1956** | **IPO2114, SCRI 4073, NCPPB 453, CFBP 1200** | - | - | - | - | - | - | - | - | - | - | - | - | - | - | - | - | - | - | - | - | - | - | - | - | - | - | - | - |
| **IFB 0145** | ***D. dianthicola*** | **Wildtype, river water, UK** | **River 1-1** | - | - | - | - | - | - | - | - | - | - | - | - | - | - | - | - | - | - | - | - | - | - | - | - | - | - | - | - |
| **SASA 2260** | ***D. dianthicola*** | **Wildtype, *Solanum tuberosum*, UK** | - | - | - | - | - | - | - | - | - | - | - | - | - | - | - | - | - | - | - | - | - | - | - | - | - | - | - | - | - |
| **IFB 0116** | ***D. paradisiaca*** | **Wildtype, *Zea mays*, Cuba, 1987** | **IPO2128, CFBP 3699** | - | - | - | - | - | - | - | - | - | - | - | - | - | - | - | - | - | - | - | - | - | - | - | - | - | - | - | - |
| **IFB 0117^T^** | ***D. paradisiaca*** | **Wildtype, Musa paradisiaca, Colombia, 1970** | **IPO2129, NCPPB 2511, CFBP 4178** | - | - | - | - | - | - | - | - | - | - | - | - | - | - | - | - | - | - | - | - | - | - | - | - | - | - | - | - |
| **IFB 0099** | ***D. solani*** | **Wildtype, *Solanum tuberosum*, Poland, 2005** | **IPO2276, A5348,**  **101A9/2005** | + | + | + | + | + | + | + | + | + | + | + | + | + | + | + | + | + | + | + | + | + | + | + | + | + | + | + | + |
| **IFB 0100** | ***D. solani*** | **Wildtype, *Solanum tuberosum*, Poland, 2005** | **IPO2277,**  **101A11/2005** | + | + | + | + | + | + | + | + | + | + | + | + | + | + | + | + | + | + | + | + | + | + | + | + | + | + | + | + |
| **IFB 0101** | ***D. solani*** | **Wildtype, *Solanum tuberosum*, Poland, 2005** | **IPO2278,**  **101A12/2005** | + | + | + | + | + | + | + | + | + | + | + | + | + | + | + | + | + | + | + | + | + | + | + | + | + | + | + | + |
| **IFB 0124** | ***D. solani*** | **Wildtype, *Solanum tuberosum*, Germany, 2008** | **Imp J-52, IPO3238** | + | + | + | + | + | + | + | + | + | + | + | + | + | + | + | + | + | + | + | + | + | + | + | + | + | + | + | + |
| **IFB 0125** | ***D. solani*** | **Wildtype, Israel** | **IPO3296, G-298** | + | + | + | + | + | + | + | + | + | + | + | + | + | + | + | + | + | + | + | + | + | + | + | + | + | + | + | + |
| **IFB 0133** | ***D. solani*** | **Wildtype, UK** | **IPO3239, 20711883** | + | + | + | + | + | + | + | + | + | + | + | + | + | + | + | + | + | + | + | + | + | + | + | + | + | + | + | + |
| **IFB 0135** | ***D. solani*** | **Wildtype, river water, Finland** | **IPO3295, Ds0432-1** | + | + | + | + | + | + | + | + | + | + | + | + | + | + | + | + | + | + | + | + | + | + | + | + | + | + | + | + |
| **IFB 0141** | ***D. solani*** | **Wildtype, *Hyacinthus* sp. the Netherlands** | **IPO2019** | + | + | + | + | + | + | + | + | + | + | + | + | + | + | + | + | + | + | + | + | + | + | + | + | + | + | + | + |
| **IFB 0158** | ***D. solani*** | **Wildtype, *Solanum tuberosum*, Poland, 2009** | **50A/1/2009. A5349** | + | + | + | + | + | + | + | + | + | + | + | + | + | + | + | + | + | + | + | + | + | + | + | + | + | + | + | + |
| **IFB 0167** | ***D. solani*** | **Wildtype, Solanum tuberosum, Poland, 2009** | **94A/10/2009** | + | + | + | + | + | + | + | + | + | + | + | + | + | + | + | + | + | + | + | + | + | + | + | + | + | + | + | + |
| **IFB 0168** | ***D. solani*** | **Wildtype, Solanum tuberosum, Poland, 2009** | **95A/10/2009** | + | + | + | + | + | + | + | + | + | + | + | + | + | + | + | + | + | + | + | + | + | + | + | + | + | + | + | + |
| **IFB 0169** | ***D. solani*** | **Wildtype, Solanum tuberosum, Poland, 2009** | **98A/10/2009** | + | + | + | + | + | + | + | + | + | + | + | + | + | + | + | + | + | + | + | + | + | + | + | + | + | + | + | + |
| **IFB 0212** | ***D. solani*** | **Wildtype, Solanum tuberosum, Poland, 2010** | **1A/1/2010** | + | + | + | + | + | + | + | + | + | + | + | + | + | + | + | + | + | + | + | + | + | + | + | + | + | + | + | + |
| **IFB 0213** | ***D. solani*** | **Wildtype, *Solanum tuberosum*, Poland, 2010** | **67A/1/2010** | + | + | + | + | + | + | + | + | + | + | + | + | + | + | + | + | + | + | + | + | + | + | + | + | + | + | + | + |
| **IFB 0223** | ***D. solani*** | **Wildtype, *Solanum tuberosum*, Germany** | **457, A5350** | + | + | + | + | + | + | + | + | + | + | + | + | + | + | + | + | + | + | + | + | + | + | + | + | + | + | + | + |
| **IPO2222^T^** | ***D. solani*** | **Wildtype, *Solanum tuberosum*, the Netherlands, 2005** | **IFB 0123, 202, A5330** | + | + | + | + | + | + | + | + | + | + | + | + | + | + | + | + | + | + | + | + | + | + | + | + | + | + | + | + |
| **IFB 0003** | ***D. zeae*** | **Wildtype, *Zea mays*, Egypt** | **IPO1271, SCRI 4078, NCPPB 1065** | - | - | - | - | - | - | - | - | - | - | - | - | - | - | - | - | - | - | - | - | - | - | - | - | - | - | - | - |
| **IFB 0051** | ***D. zeae*** | **Wildtype, *Solanum tuberosum*, Peru** | **CIP 366** | - | - | - | - | - | - | - | - | - | - | - | - | - | - | - | - | - | - | - | - | - | - | - | - | - | - | - | - |
| **IFB 0087** | ***D. zeae*** | **Wildtype, *Solanum tuberosum*, Australia** | **IPO647** | - | - | - | - | - | - | - | - | - | - | - | - | - | - | - | - | - | - | - | - | - | - | - | - | - | - | - | - |
| **IFB 0110^T^** | ***D. zeae*** | **Wildtype, Ananas comosus, Malaysia, 1961** | **IPO2121, NCPPB 1121, CFBp 1278** | - | - | - | - | - | - | - | - | - | - | - | - | - | - | - | - | - | - | - | - | - | - | - | - | - | - | - | - |
| **IFB 0189** | ***D. zeae*** | **Wildtype, river water, Poland** | **4034 (15)** | - | - | - | - | - | - | - | - | - | - | - | - | - | - | - | - | - | - | - | - | - | - | - | - | - | - | - | - |
| **IPO 161** | ***P. atrosepticum*** | **wildtype, *Solanum tuberosum*, the Netherlands** | - | - | - | - | - | - | - | - | - | - | - | - | - | - | - | - | - | - | - | - | - | - | - | - | - | - | - | - | - |
| **IFB 5110** | ***P. atrosepticum*** | **Wildtype, *Solanum tuberosum*, Poland, 2005** | **UGCA49** | - | - | - | - | - | - | - | - | - | - | - | - | - | - | - | - | - | - | - | - | - | - | - | - | - | - | - | - |
| **IFB 5028** | ***P. atrosepticum*** | **Wildtype, *Solanum tuberosum*, Poland, 2005** | **UGCA64** | - | - | - | - | - | - | - | - | - | - | - | - | - | - | - | - | - | - | - | - | - | - | - | - | - | - | - | - |
| **IFB 5007** | ***P. atrosepticum*** | **wildtype, *Solanum tuberosum*, Peru, 1978** | **SCRI 85** | - | - | - | - | - | - | - | - | - | - | - | - | - | - | - | - | - | - | - | - | - | - | - | - | - | - | - | - |
| **IFB 5012** | ***P. atrosepticum*** | **wildtype, *Solanum tuberosum*, Israel, 1955** | **SCRI 1054** | - | - | - | - | - | - | - | - | - | - | - | - | - | - | - | - | - | - | - | - | - | - | - | - | - | - | - | - |
| **IFB 5013** | ***P. atrosepticum*** | **wildtype, *Solanum tuberosum*, UK, 1977** | **SCRI 1055** | - | - | - | - | - | - | - | - | - | - | - | - | - | - | - | - | - | - | - | - | - | - | - | - | - | - | - | - |
| **IFB 5015** | ***P. atrosepticum*** | **wildtype, *Solanum tuberosum*, UK, 1977** | **SCRI 1092** | - | - | - | - | - | - | - | - | - | - | - | - | - | - | - | - | - | - | - | - | - | - | - | - | - | - | - | - |
| **IFB 5024** | ***P. atrosepticum*** | **wildtype, *Solanum tuberosum*, UK** | **IPO 1073** | - | - | - | - | - | - | - | - | - | - | - | - | - | - | - | - | - | - | - | - | - | - | - | - | - | - | - | - |
| **IFB 5030** | ***P. atrosepticum*** | **wildtype, *Solanum tuberosum,* Sweden** | **IPO 1155** | - | - | - | - | - | - | - | - | - | - | - | - | - | - | - | - | - | - | - | - | - | - | - | - | - | - | - | - |
| **IFB 5033** | ***P. atrosepticum*** | **wildtype, bulk soil, UK** | **IPO 1187** | - | - | - | - | - | - | - | - | - | - | - | - | - | - | - | - | - | - | - | - | - | - | - | - | - | - | - | - |
| **IFB 5038** | ***P. atrosepticum*** | **wildtype, *Solanum lycopersicum*, Fraqnce, 1973** | **IPO 1336** | - | - | - | - | - | - | - | - | - | - | - | - | - | - | - | - | - | - | - | - | - | - | - | - | - | - | - | - |
| **IFB 5044** | ***P. atrosepticum*** | **wildtype, *Solanum tuberosum*, Germany** | **IPO 1322** | - | - | - | - | - | - | - | - | - | - | - | - | - | - | - | - | - | - | - | - | - | - | - | - | - | - | - | - |
| **IFB 5045** | ***P. atrosepticum*** | **wildtype, *Solanum tuberosum*, USA** | **IPO 1321** | - | - | - | - | - | - | - | - | - | - | - | - | - | - | - | - | - | - | - | - | - | - | - | - | - | - | - | - |
| **IFB 5102** | ***P. atrosepticum*** | **wildtype, *Solanum tuberosum*, UK** | **SCRI 1043** | - | - | - | - | - | - | - | - | - | - | - | - | - | - | - | - | - | - | - | - | - | - | - | - | - | - | - | - |
| **IFB 5105** | ***P. atrosepticum*** | **wildtype, *Solanum tuberosum*, the Netherlands** | **SCRI 1091** | - | - | - | - | - | - | - | - | - | - | - | - | - | - | - | - | - | - | - | - | - | - | - | - | - | - | - | - |
| **IFB 5106** | ***P. atrosepticum*** | **wildtype, *Solanum tuberosum*, UK, 1982** | **SCRI 1113** | - | - | - | - | - | - | - | - | - | - | - | - | - | - | - | - | - | - | - | - | - | - | - | - | - | - | - | - |
| **IFB 5230** | ***P. atrosepticum*** | **wildtype, *Solanum tuberosum*, Peru, 1977** | **SCRI 176** | - | - | - | - | - | - | - | - | - | - | - | - | - | - | - | - | - | - | - | - | - | - | - | - | - | - | - | - |
| **IFB 5237** | ***P. atrosepticum*** | **wildtype, *Solanum tuberosum*, Peru, 1977** | **SCRI 174** | - | - | - | - | - | - | - | - | - | - | - | - | - | - | - | - | - | - | - | - | - | - | - | - | - | - | - | - |
| **IFB 5309** | ***P. atrosepticum*** | **wildtype, *Solanum tuberosum*, UK, 1977** | **SCRI 103** | - | - | - | - | - | - | - | - | - | - | - | - | - | - | - | - | - | - | - | - | - | - | - | - | - | - | - | - |
| **IFB 5374** | ***P. atrosepticum*** | **Wildtype, *Solanum tuberosum*, Poland, 2011** | **-** | - | - | - | - | - | - | - | - | - | - | - | - | - | - | - | - | - | - | - | - | - | - | - | - | - | - | - | - |
| **IFB 5377** | ***P. atrosepticum*** | **Wildtype, *Solanum tuberosum*, Poland, 2005** | **-** | - | - | - | - | - | - | - | - | - | - | - | - | - | - | - | - | - | - | - | - | - | - | - | - | - | - | - | - |
| **IFB 5380** | ***P. atrosepticum*** | **Wildtype, *Solanum tuberosum*, Poland, 2009** | **-** | - | - | - | - | - | - | - | - | - | - | - | - | - | - | - | - | - | - | - | - | - | - | - | - | - | - | - | - |
| **IFB 5399^T^** | ***P. atrosepticum*** | **Wildtype, *Solanum tuberosum*, UK, 1969** | **LMG 2386, ATCC 33260, NCPPB 549** | - | - | - | - | - | - | - | - | - | - | - | - | - | - | - | - | - | - | - | - | - | - | - | - | - | - | - | - |
| **IFB 5037** | ***P. atrosepticum*** | **Wildtype, *Solanum tuberosum*, France, 1969** | **IPO 1319** | - | - | - | - | - | - | - | - | - | - | - | - | - | - | - | - | - | - | - | - | - | - | - | - | - | - | - | - |
| **IFB 5001** | ***P. atrosepticum*** | **Wildtype, *Solanum tuberosum*, USA** | **SCRI 16** | - | - | - | - | - | - | - | - | - | - | - | - | - | - | - | - | - | - | - | - | - | - | - | - | - | - | - | - |
| **IFB 5231** | ***P. atrosepticum*** | **wildtype, *Solanum tuberosum*, Peru, 1978** | **SCRI 178** | - | - | - | - | - | - | - | - | - | - | - | - | - | - | - | - | - | - | - | - | - | - | - | - | - | - | - | - |
| **Pcb JJ56** | ***P. carotovorum subsp. brasiliense*** | **Wildtype, *Solanum tuberosum*, South Africa** | **-** | - | - | - | - | - | - | - | - | - | - | - | - | - | - | - | - | - | - | - | - | - | - | - | - | - | - | - | - |
| **LMG**  **21371^T^** | ***P. carotovorum subsp. brasiliense*** | **wildtype, *Solanum tuberosum*, 1999** | **CFBP 6617,**  **Ecbr 212,**  **IBSBF 1692** | - | - | - | - | - | - | - | - | - | - | - | - | - | - | - | - | - | - | - | - | - | - | - | - | - | - | - | - |
| **Ecc 71** | ***P. carotovorum subsp. carotovorum*** | **wildtype, *Solanum tuberosum*, 1987** | **-** | + | + | + | + | + | + | + | + | + | + | + | + | + | + | + | + | + | + | + | + | + | + | + | + | + | + | + | + |
| **IFB 5127** | ***P. carotovorum subsp. carotovorum*** | **wildtype, *Solanum tuberosum*, Tasmania, 1973** | **SCRI 156** | + | + | - | - | - | - | - | - | - | - | - | - | - | - | - | - | - | - | - | - | - | - | - | - | - | - | - | - |
| **IFB 5123** | ***P. carotovorum subsp. carotovorum*** | **wildtype, *Solanum tuberosum*, Tasmania, 1973** | **SCRI 147** | + | + | + | + | + | + | + | + | + | + | + | + | + | + | + | + | + | + | + | + | + | + | + | + | + | + | + | + |
| **IFB 5129** | ***P. carotovorum subsp. carotovorum*** | **wild type, fly, UK, 1972** | **SCRI 169** | + | + | + | + | + | + | + | + | + | + | + | + | + | + | + | + | + | + | + | + | + | + | + | + | + | + | + | + |
| **IFB 5126** | ***P. carotovorum subsp. carotovorum*** | **wildtype, *Solanum tuberosum*, Tasmania, 1973** | **SCRI 154** | + | + | + | + | + | + | + | + | + | + | + | + | + | + | + | + | + | + | + | + | + | + | + | + | + | + | + | + |
| **IFB 5122** | ***P. carotovorum subsp. carotovorum*** | **wildtype, *Solanum tuberosum*, Tasmania, 1973** | **SCRI 146** | + | + | + | + | + | + | + | + | + | + | + | + | + | + | + | + | + | + | + | + | + | + | + | + | + | + | + | + |
| **IFB 5186** | ***P. carotovorum subsp. carotovorum*** | **wildtype, *Solanum tuberosum*, Tasmania** | **SCRI 143** | + | + | + | + | + | + | + | + | + | + | + | + | + | + | + | + | + | + | + | + | + | + | + | + | + | + | + | + |
| **IFB 5187** | ***P. carotovorum subsp. carotovorum*** | **wildtype, *Solanum tuberosum*, Tasmania** | **SCRI 144** | + | + | + | + | + | + | + | + | + | + | + | + | + | + | + | + | + | + | + | + | + | + | + | + | + | + | + | + |
| **IFB 5125** | ***P. carotovorum subsp. carotovorum*** | **wildtype, *Solanum tuberosum*, Tasmania, 1973** | **SCRI 152** | + | + | + | + | + | + | + | + | + | + | + | + | + | + | + | + | + | + | + | + | + | + | + | + | + | + | + | + |
| **IFB 5118** | ***P. carotovorum subsp. carotovorum*** | **wildtype, *Solanum tuberosum*, USA** | **SCRI 136** | + | + | + | + | + | + | + | + | + | + | + | + | + | + | + | + | + | + | + | + | + | + | + | + | + | + | + | + |
| **IFB 5124** | ***P. carotovorum subsp. carotovorum*** | **wildtype, *Solanum tuberosum*, Tasmania, 1973** | **SCRI 149** | + | + | + | + | + | + | + | + | + | + | + | + | + | + | + | + | + | + | + | + | + | + | + | + | + | + | + | + |
| **IFB 5188** | ***P. carotovorum subsp. carotovorum*** | **wildtype, *Solanum tuberosum*, Tasmania, 1973** | **SCRI 148** | + | + | + | + | + | + | + | + | + | + | + | + | + | + | + | + | + | + | + | + | + | + | + | + | + | + | + | + |
| **IFB 5119** | ***P. carotovorum subsp. carotovorum*** | **wildtype, *Solanum tuberosum*, USA** | **SCRI 138** | + | + | + | + | + | + | + | + | + | + | + | + | + | + | + | + | + | + | + | + | + | + | + | + | + | + | + | + |
| **IFB 5128** | ***P. carotovorum subsp. carotovorum*** | **wildtype, *Solanum tuberosum*, Tasmania, 1970** | **SCRI 162** | - | - | - | - | - | - | - | - | - | - | - | - | - | - | - | - | - | - | - | - | - | - | - | - | - | - | - | - |
| **IFB 5190** | ***P. carotovorum subsp. carotovorum*** | **wildtype, *Solanum tuberosum*, Tasmania, 1970** | **SCRI 164** | + | + | + | + | + | + | + | + | + | + | + | + | + | + | + | + | + | + | + | + | + | + | + | + | + | + | + | + |
| **IFB 5120** | ***P. carotovorum subsp. carotovorum*** | **wildtype, *Solanum tuberosum*, USA** | **SCRI 139** | + | + | + | + | + | + | + | + | + | + | + | + | + | + | + | + | + | + | + | + | + | + | + | + | + | + | + | + |
| **IFB 5189** | ***P. carotovorum subsp. carotovorum*** | **wildtype, *Solanum tuberosum*, Tasmania, 1973** | **SCRI 159** | + | + | + | + | + | + | + | + | + | + | + | + | + | + | + | + | + | + | + | + | + | + | + | + | + | + | + | + |
| **IPO 202** | ***P. carotovorum subsp. carotovorum*** | **Wildtype, *Solanum tuberosum*, the Netherlands, 1974** | **-** | + | + | + | + | + | + | + | + | + | + | + | + | + | + | + | + | + | + | + | + | + | + | + | + | + | + | + | + |
| **IPO 454** | ***P. carotovorum subsp. carotovorum*** | **Wildtype, *Cichorium endivia*, the Netherlands, 1978** | **-** | - | - | - | - | - | - | - | - | - | - | - | - | - | - | - | - | - | - | - | - | - | - | - | - | - | - | - | - |
| **IPO 167** | ***P. carotovorum subsp. carotovorum*** | **Wildtype, *Cichorium intybus*, the Netherlands, 1974** | **-** | - | - | - | - | - | - | - | - | - | - | - | - | - | - | - | - | - | - | - | - | - | - | - | - | - | - | - | - |
| **IPO 280** | ***P. carotovorum subsp. carotovorum*** | **Wildtype, *Brassica oleracea*, UK** | **CL43/8** | - | - | - | - | - | - | - | - | - | - | - | - | - | - | - | - | - | - | - | - | - | - | - | - | - | - | - | - |
| **IPO 495** | ***P. carotovorum subsp. carotovorum*** | **Wildtype, *Brassica oleracea*, the Netherlands, 1978** | **-** | + | + | + | + | + | + | + | + | + | + | + | + | + | + | + | + | + | + | + | + | + | + | + | + | + | + | + | + |
| **IPO 497** | ***P. carotovorum subsp. carotovorum*** | **Wildtype, *Brassica oleracea*, the Netherlands, 1978** | **-** | - | - | - | - | - | - | - | - | - | - | - | - | - | - | - | - | - | - | - | - | - | - | - | - | - | - | - | - |
| **IPO 554** | ***P. carotovorum subsp. carotovorum*** | **Wildtype, *Helianthus* *annuus*, the Netherlands, 1978** | **-** | + | + | + | + | + | + | + | + | + | + | + | + | + | + | + | + | + | + | + | + | + | + | + | + | + | + | + | + |
| **IFB 5395** | ***P. wasabiae*** | **Wildtype, *Solanum tuberosum*, Finland** | **SCC3193** | - | - | - | - | - | - | - | - | - | - | - | + | - | - | - | - | - | - | - | - | + | - | - | - | - | - | - | - |
| **IFB 5303** | ***P. wasabiae*** | **Wildtype, *Armoracia rusticana*, Japan** | **SCRI 488** | - | - | - | - | - | - | - | - | - | - | - | + | - | - | - | - | - | - | - | - | + | - | - | - | - | - | - | - |
| **IFB 5310** | ***P. wasabiae*** | **Wildtype, *Solanum tuberosum*, USA** | **SCRI 140** | - | - | - | - | - | - | - | - | - | - | - | + | - | - | - | - | - | - | - | - | + | - | - | - | - | - | - | - |
| **IFB 5309** | ***P. wasabiae*** | **Wildtype, Solanum tuberosum, UK, 1977** | **SCRI 103** | - | - | - | - | - | - | - | - | - | - | - | + | - | - | - | - | - | - | - | - | + | - | - | - | - | - | - | - |
| **IPO 139** | ***P. wasabiae*** | **Wildtype, *Solanum tuberosum*, the Netherlands, 1973** | **-** | - | - | - | - | - | - | - | - | - | - | - | + | - | - | - | - | - | - | - | - | + | - | - | - | - | - | - | - |
| **IPO 222** | ***P. wasabiae*** | **Wildtype, *Solanum tuberosum*, the Netherlands, 1975** | **-** | - | - | - | - | - | - | - | - | - | - | - | + | - | - | - | - | - | - | - | - | + | - | - | - | - | - | - | - |

^a^ (+) indicates lysis of bacterial cells (plaque formation), (-) indicates lack of lysis of bacterial cells (no plaque formation)

^T^ - type strain

Phages (φPD10.3 and φPD23.1) used for further study are highlighted in grey
