# Supplementary material for: Genomic, Proteomic and Morphological Characterization of Two Novel Broad Host Lytic Bacteriophages ΦPD10.3 and ΦPD23.1 Infecting Pectinolytic Pectobacterium spp. and Dickeya spp
Source: PLoS One. 2015 Mar 24;10(3):e0119812. doi: 10.1371/journal.pone.0119812 (PMC4372400; doi:10.1371/journal.pone.0119812)
Supplement: S2 Table — ORFs coding for hypothetical proteins and/or coding for conserved hypothetical proteins are not shown in the table. (DOCX) [file pone.0119812.s003.docx]

**S3 Table.** Summary of the 84 (φPD10.3) and 88 (φPD23.1.1) ORFs (PEGs) with predicted, assigned function. ORFs coding for hypothetical proteins and/or coding for conserved hypothetical proteins are not shown in the table.

| **No.** | **Locus taq (ORF)** | **5' end** | **3' end** | **Predicted function (Pfam) ^a^** | **Length (aa) ^b^** | **Taxon** | **% identity** | **P - value** |
| --- | --- | --- | --- | --- | --- | --- | --- | --- |
| ***Bacteriophage φPD10.3 ORFs (PEGs) with predicted functions*** | | | | | | | | |
| **1** | PD10.3_001 | 564 | 13 | virus neck family protein | 183 | unclassified *Myoviridae* | 98.3 | 5e-99 |
| **2** | PD10.3_003 | 1616 | 864 | putative gp13 neck protein | 250 | *Salmonella* phage SKML-39 | 95.6 | 1e-126 |
| **3** | PD10.3_007 | 8743 | 7097 | putative tailspike protein | 548 | *Enterobactertia* phage K1F | 49.1 | 4e-06 |
| **4** | PD10.3_008 | 11675 | 8799 | putative tailspike protein | 958 | *Salmonella* phage SKML-39 | 52 | 3e-88 |
| **5** | PD10.3_010 | 15336 | 12277 | right handed beta helix region family protein | 1019 | *Dickeya* phage vB_DsoM_LIMEstone1 | 71.6 | 0 |
| **6** | PD10.3_013 | 19219 | 17441 | baseplate J-like family protein | 592 | *Enterobacteria* phage T4 | 25 | 4e-24 |
| **7** | PD10.3_027 | 28275 | 29708 | DNA polymerase family B, exonuclease domain protein | 477 | *Enterobacteria* phage RB69 | 29.7 | 3e-24 |
| **8** | PD10.3_030 | 30829 | 31308 | DNA polymerase domain protein | 159 | *Dickeya* phage vB_DsoM_LIMEstone1 | 98.7 | 4e-86 |
| **9** | PD10.3_031 | 32184 | 33317 | DNA polymerase B family protein | 377 | *Enterobacteria* phage RB69 | 33.7 | 3e-49 |
| **10** | PD10.3_033 | 33718 | 34497 | 5' nucleotidase, deoxy family protein | 259 | *Dickeya* phage vB_DsoM_LIMEstone1 | 100 | 1e-143 |
| **11** | PD10.3_054 | 48191 | 49000 | ig-like virion protein | 269 | *Serratia* phage KSP90 | 45.1 | 1e-43 |
| **12** | PD10.3_060 | 51052 | 51825 | putative HNH homing endonuclease domain protein | 257 | *Dickeya* phage vB_DsoM_LIMEstone1 | 100 | 1e-147 |
| **13** | PD10.3_062 | 53776 | 54297 | AP2 domain protein | 173 | *Dickeya* phage vB_DsoM_LIMEstone1 | 100 | 1e-102 |
| **14** | PD10.3_063 | 54294 | 55628 | DNA gyrase/topoisomerase IV, subunit A family protein | 444 | *Schizosaccharomyces pombe* 972h- | 32.6 | 7e-49 |
| **15** | PD10.3_072 | 57976 | 58482 | cytidine and deoxycytidylate deaminase zinc-binding region family protein | 168 | *Dickeya* phage vB_DsoM_LIMEstone1 | 100 | 2e-91 |
| **16** | PD10.3_074 | 59680 | 59063 | putative gp4 head completion protein | 205 | *Dickeya* phage vB_DsoM_LIMEstone1 | 99 | 1e-106 |
| **17** | PD10.3_076 | 60469 | 61437 | putative gp48 T4-like baseplate tail tube cap | 322 | unclassified *Myoviridae* | 97.5 | 0 |
| **18** | PD10.3_078 | 63128 | 63682 | base plate wedge 53 family protein | 184 | *Dickeya* phage vB_DsoM_LIMEstone1 | 100 | 1e-103 |
| **19** | PD10.3_080 | 67693 | 67028 | loader of gp41 DNA helicase family protein | 221 | *Dickeya* phage vB_DsoM_LIMEstone1 | 98.6 | 1e-123 |
| **20** | PD10.3_083 | 69361 | 67937 | ATP dependent DNA ligase domain protein | 474 | *Salmonella* phage STML-13-1 | 81.4 | 0 |
| **21** | PD10.3_086 | 71755 | 71153 | NUMOD3 motif family protein | 200 | *Dickeya* phage vB_DsoM_LIMEstone1 | 99.5 | 1e-115 |
| **22** | PD10.3_089 | 73909 | 72482 | dnaB-like helicase C terminal domain protein | 475 | *Myoviridae* | 31.3 | 4e-66 |
| **23** | PD10.3_091 | 75307 | 74222 | recA bacterial DNA recombination family protein | 361 | *Enterobacteria* phage T4 | 36.2 | 6e-61 |
| **24** | PD10.3_093 | 76388 | 75834 | dUTPase family protein | 184 | *Dickeya* phage vB_DsoM_LIMEstone1 | 93.5 | 4e-98 |
| **25** | PD10.3_095 | 77997 | 76951 | thymidylate synthase family protein | 348 | *Bacillus* phage SPO1 | 37.7 | 3e-22 |
| **26** | PD10.3_099 | 81463 | 80762 | putative gp2 DNA end protector protein | 233 | *Dickeya* phage vB_DsoM_LIMEstone1 | 100 | 1e-137 |
| **27** | PD10.3_100 | 81518 | 82462 | T4-like virus tail tube gp19 family protein | 314 | *Dickeya* phage vB_DsoM_LIMEstone1 | 99.4 | 1e-176 |
| **28** | PD10.3_101 | 83535 | 82489 | gp32 DNA binding like family protein | 348 | *Enterobacteria* phage RB69 | 29.7 | 2e-14 |
| **29** | PD10.3_103 | 84126 | 83881 | putative gp33 T4-like late promoter transcription accessory protein | 81 | *Dickeya* phage vB_DsoM_LIMEstone1 | 100 | 8e-36 |
| **30** | PD10.3_104 | 84364 | 84119 | regulatory , FmdB family domain protein | 81 | *Dickeya* phage vB_DsoM_LIMEstone1 | 100 | 7e-41 |
| **31** | PD10.3_109 | 86348 | 87154 | T4 bacteriophage base plate family protein | 268 | *Dickeya* phage vB_DsoM_LIMEstone1 | 99.6 | 1e-139 |
| **32** | PD10.3_113 | 89348 | 89728 | lysozyme family protein | 126 | unclassified Myoviridae | 100 | 6e-67 |
| **33** | PD10.3_120 | 97402 | 96608 | putative peptidoglycan binding domain protein | 264 | *Dickeya* phage vB_DsoM_LIMEstone1 | 100 | 1e-148 |
| **34** | PD10.3_125 | 99766 | 98906 | putative gp61 DNA primase subunit | 286 | *Dickeya* phage vB_DsoM_LIMEstone1 | 100 | 1e-156 |
| **35** | PD10.3_135 | 106176 | 105562 | endoribonuclease RegB T4-bacteriophage encoded family protein | 204 | *Dickeya* phage vB_DsoM_LIMEstone1 | 100 | 1e-103 |
| **36** | PD10.3_139 | 110268 | 109153 | putative gp47 recombination protein subunit | 371 | *Salmonella* phage SKML-39 | 97 | 0 |
| **37** | PD10.3_140 | 110996 | 110268 | putative gp55 T4-like sigma factor involved in late transcription | 242 | *Dickeya* phage vB_DsoM_LIMEstone1 | 100 | 1e-126 |
| **38** | PD10.3_141 | 111535 | 111008 | RNase H family protein | 175 | *Dickeya* phage vB_DsoM_LIMEstone1 | 100 | 2e-99 |
| **39** | PD10.3_143 | 114023 | 112305 | type III restriction enzyme, res subunit | 572 | *Salmonella* phage PhiSH19 | 85.7 | 0 |
| **40** | PD10.3_145 | 114431 | 114153 | bacterial DNA-binding family protein | 92 | *Dickeya* phage vB_DsoM_LIMEstone1 | 100 | 3e-43 |
| **41** | PD10.3_154 | 118836 | 118381 | endonuclease V | 151 | *Enterobacteria* phage T4 | 42.6 | 1e-11 |
| **42** | PD10.3_156 | 119583 | 119260 | putative acyl carrier protein | 107 | *Dickeya* phage vB_DsoM_LIMEstone1 | 100 | 2e-54 |
| **43** | PD10.3_166 | 127292 | 126828 | translation repressor domain protein | 154 | Viruses | 41.3 | 4e-19 |
| **44** | PD10.3_167 | 127744 | 127322 | putative gp62 clamp loader subunit DNA polymerase accessory protein | 140 | *Dickeya* phage vB_DsoM_LIMEstone1 | 100 | 1e-75 |
| **45** | PD10.3_168 | 128507 | 127749 | DNA polymerase III, delta subunit | 252 | *Saccharomyces cerevisiae* | 32 | 3e-12 |
| **46** | PD10.3_169 | 129196 | 128504 | putative gp45 sliding clamp DNA polymerase accessory protein | 230 | *Dickeya* phage vB_DsoM_LIMEstone1 | 92 | 2e-87 |
| **47** | PD10.3_171 | 130279 | 129707 | putative gp45 sliding clamp DNA polymerase accessory protein | 190 | unclassified *Myoviridae* | 97.9 | 2e-90 |
| **48** | PD10.3_172 | 131331 | 130999 | putative uvsW RNA-and DNA-helicase | 110 | Viruses | 47.1 | 4e-13 |
| **49** | PD10.3_175 | 132558 | 132205 | RNA-DNA and DNA-DNA helicase UvsW domain protein | 117 | *Dickeya* phage vB_DsoM_LIMEstone1 | 99.5 | 4e-58 |
| **50** | PD10.3_177 | 134172 | 133426 | PD-(D/E)XK nuclease superfamily protein | 248 | *Dickeya* phage vB_DsoM_LIMEstone1 | 100 | 1e-141 |
| **51** | PD10.3_178 | 134627 | 134172 | recombination, repair and ssDNA binding UvsY family protein | 151 | *Dickeya* phage vB_DsoM_LIMEstone1 | 100 | 9e-83 |
| **52** | PD10.3_179 | 135170 | 134670 | putative gp3-tail completion | 166 | *Dickeya* phage vB_DsoM_LIMEstone1 | 100 | 3e-82 |
| **53** | PD10.3_189 | 140167 | 139319 | NUMOD3 motif family protein | 282 | Viruses | 64.4 | 1e-114 |
| **54** | PD10.3_190 | 141597 | 140269 | major capsid protein | 442 | unclassified *Myoviridae* | 83.9 | 0 |
| **55** | PD10.3_191 | 142561 | 141689 | putative gp22 prohead core protein | 290 | *Dickeya* phage vB_DsoM_LIMEstone1 | 100 | 1e-101 |
| **56** | PD10.3_192 | 143272 | 142607 | prohead core protease family protein | 221 | *Shigella* phage phiSboM-AG3 | 99.1 | 1e-122 |
| **57** | PD10.3_195 | 145496 | 143805 | bacteriophage T4-like capsid assembly family protein | 563 | *Salmonella* phage SKML-39 | 97.3 | 0 |
| **58** | PD10.3_196 | 146097 | 145564 | tail tube protein | 177 | unclassified *Myoviridae* | 87.5 | 2e-81 |
| **59** | PD10.3_198 | 150103 | 148205 | phage tail sheath family protein | 632 | *Enterobacteria* phage T4 | 32.6 | 6e-40 |
| **60** | PD10.3_199 | 152348 | 150156 | pretoxin HINT domain protein | 730 | *Enterobacteria* phage RB49 | 35.9 | 1e-54 |
| **61** | PD10.3_201 | 153895 | 153194 | terminase DNA packaging enzyme family protein | 233 | *Dickeya* phage vB_DsoM_LIMEstone1 | 100 | 1e-117 |
| **62** | PD10.3_202 | 154593 | 153898 | putative gp15 proximal tail sheath stabilization protein | 231 | *Dickeya* phage vB_DsoM_LIMEstone1 | 100 | 1e-134 |
| **63** | PD10.3_203 | 155246 | 154596 | virus neck family protein | 216 | *Dickeya* phage vB_DsoM_LIMEstone1 | 98.1 | 1e-121 |
| **64** | PD10.3_204 | 155305 | 156012 | meiotically up-regulated family protein | 235 | *Dickeya* phage vB_DsoM_LIMEstone1 | 100 | 1e-138 |
| **65** | PD10.3_212 | 2642 | 2493 | major capsid domain protein | 49 | unclassified *Myoviridae* | 83 | 1e-16 |
| **66** | PD10.3_213 | 3588 | 3076 | helix-turn-helix domain protein | 170 | *Dickeya* phage vB_DsoM_LIMEstone1 | 98.2 | 5e-87 |
| **67** | PD10.3_221 | 8812 | 8465 | gp48 T4-like baseplate tail tube cap domain protein | 115 | unclassified *Myoviridae* | 94.6 | 2e-53 |
| **68** | PD10.3_228 | 11743 | 11886 | gp59 T4-like loader of gp41 DNA helicase domain protein | 47 | *Salmonella* phage SKML-39 | 93.3 | 6e-18 |
| **69** | PD10.3_231 | 13260 | 13021 | base plate wedge 53 family protein | 79 | *Dickeya* phage vB_DsoM_LIMEstone1 | 97.6 | 2e-38 |
| **70** | PD10.3_232 | 13634 | 13287 | RIIB domain protein | 115 | *Shigella* phage phiSboM-AG3 | 90.5 | 3e-55 |
| **71** | PD10.3_234 | 14898 | 14389 | DNA gyrase/topoisomerase IV, subunit A family protein | 169 | *Dickeya* phage vB_DsoM_LIMEstone1 | 92.9 | 4e-93 |
| **72** | PD10.3_235 | 15122 | 14919 | DNA gyrase/topoisomerase IV, subunit A family protein | 67 | *Escherichia* phage ECML-4 | 82.8 | 3e-24 |
| **73** | PD10.3_236 | 15363 | 15124 | DNA topoisomerase 2 domain protein | 79 | Viruses | 87 | 2e-23 |
| **74** | PD10.3_238 | 18076 | 16430 | putative tailspike protein | 548 | *Dickeya* phage vB_DsoM_LIMEstone1 | 100 | 0 |
| **75** | PD10.3_239 | 18646 | 18131 | tailspike domain protein | 171 | *Dickeya* phage vB_DsoM_LIMEstone1 | 100 | 1e-92 |
| **76** | PD10.3_240 | 20310 | 18796 | hemolysin-type calcium-binding domain protein | 504 | *Salmonella* phage SKML-39 | 83.2 | 0 |
| **77** | PD10.3_244 | 22906 | 24339 | RIIA domain protein | 477 | *Shigella* phage phiSboM-AG3 | 93.7 | 0 |
| **78** | PD10.3_252 | 30454 | 30810 | gp30 DNA ligase domain protein | 118 | *Salmonella* phage SKML-39 | 93.5 | 3e-56 |
| **79** | PD10.3_254 | 32789 | 33157 | gp44 clamp loader subunit DNA polymerase accessory domain protein | 122 | *Dickeya* phage vB_DsoM_LIMEstone1 | 96.7 | 1e-62 |
| **80** | PD10.3_255 | 33162 | 33584 | putative gp62 clamp loader subunit DNA polymerase accessory protein | 140 | *Dickeya* phage vB_DsoM_LIMEstone1 | 97.9 | 2e-73 |
| **81** | PD10.3_256 | 33614 | 34078 | translation repressor domain protein | 154 | Viruses | 41.3 | 4e-19 |
| **82** | PD10.3_261 | 37717 | 38430 | gp61 DNA primase subunit domain protein | 237 | unclassified *Myoviridae* | 94.9 | 1e-126 |
| **83** | PD10.3_263 | 38861 | 39403 | dUTPase family protein | 180 | *Dickeya* phage vB_DsoM_LIMEstone1 | 98.3 | 1e-98 |
| **84** | PD10.3_264 | 39552 | 40106 | gp5 baseplate hub subunit and tail lysozyme domain protein | 184 | *Enterobacteria* phage T4 | 38 | 4e-10 |
| ***Bacteriophage φPD23.1 ORFs (PEGs) with predicted functions*** | | | | | | | | |
| **1** | PD23.1_004 | 4364 | 3699 | loader of gp41 DNA helicase family protein | 221 | *Dickeya* phage vB_DsoM_LIMEstone1 | 96.8 | 1e-121 |
| **2** | PD23.1_007 | 6032 | 4608 | ATP dependent DNA ligase domain protein | 474 | *Salmonella* phage STML-13-1 | 82.5 | 0 |
| **3** | PD23.1_011 | 8424 | 7822 | NUMOD3 motif family protein | 200 | *Dickeya* phage vB_DsoM_LIMEstone1 | 99.5 | 1e-114 |
| **4** | PD23.1_014 | 10580 | 9153 | dnaB-like helicase C terminal domain protein | 475 | *Myoviridae* | 31.3 | 4e-66 |
| **5** | PD23.1_018 | 13061 | 12507 | dUTPase family protein | 184 | *Dickeya* phage vB_DsoM_LIMEstone1 | 99.5 | 1e-103 |
| **6** | PD23.1_020 | 14670 | 13624 | thymidylate synthase family protein | 348 | *Bacillus* phage SPO1 | 37.7 | e3-22 |
| **7** | PD23.1_024 | 18136 | 17435 | putative gp2 DNA end protector protein | 233 | *Dickeya* phage vB_DsoM_LIMEstone1 | 99.6 | 1e-136 |
| **8** | PD23.1_025 | 18191 | 19135 | T4-like virus tail tube gp19 family protein | 314 | *Dickeya* phage vB_DsoM_LIMEstone1 | 99.7 | 1e-177 |
| **9** | PD23.1_026 | 20208 | 19162 | gp32 DNA binding like family protein | 348 | *Enterobacteria* phage RB69 | 29.7 | 2e-14 |
| **10** | PD23.1_028 | 20799 | 20554 | putative gp33 T4-like late promoter transcription accessory protein | 81 | *Dickeya* phage vB_DsoM_LIMEstone1 | 100 | 8e-36 |
| **11** | PD23.1_029 | 21037 | 20792 | regulatory , FmdB family domain protein | 81 | *Dickeya* phage vB_DsoM_LIMEstone1 | 100 | 7e-41 |
| **12** | PD23.1_034 | 23021 | 23827 | T4 bacteriophage base plate family protein | 268 | *Dickeya* phage vB_DsoM_LIMEstone1 | 99.6 | 1e-139 |
| **13** | PD23.1_037 | 26021 | 26401 | lysozyme family protein | 126 | unclassified *Myoviridae* | 100 | 6e-67 |
| **14** | PD23.1_045 | 33766 | 32972 | putative peptidoglycan binding domain protein | 264 | *Dickeya* phage vB_DsoM_LIMEstone1 | 99.6 | 1e-147 |
| **15** | PD23.1_051 | 36130 | 35273 | putative gp61 DNA primase subunit | 285 | unclassified *Myoviridae* | 94.3 | 1e-150 |
| **16** | PD23.1_056 | 38878 | 38309 | putative phage associated protein | 189 | *Dickeya* phage vB_DsoM_LIMEstone1 | 100 | 1e-105 |
| **17** | PD23.1_057 | 40530 | 38935 | putative phage associated protein | 531 | *Dickeya* phage vB_DsoM_LIMEstone1 | 99.7 | 0 |
| **18** | PD23.1_061 | 42540 | 41926 | endoribonuclease RegB T4-bacteriophage encoded family protein | 204 | *Dickeya* phage vB_DsoM_LIMEstone1 | 97.5 | 1e-100 |
| **19** | PD23.1_065 | 46572 | 45457 | putative gp47 recombination protein subunit | 371 | *Salmonella* phage SKML-39 | 97 | 0 |
| **20** | PD23.1_066 | 47300 | 46572 | putative gp55 T4-like sigma factor involved in late transcription | 242 | *Dickeya* phage vB_DsoM_LIMEstone1 | 100 | 1e-126 |
| **21** | PD23.1_067 | 47839 | 47312 | RNase H family protein | 175 | *Dickeya* phage vB_DsoM_LIMEstone1 | 100 | 2e-99 |
| **22** | PD23.1_070 | 50775 | 50497 | bacterial DNA-binding family protein | 92 | *Dickeya* phage vB_DsoM_LIMEstone1 | 100 | 3e-43 |
| **23** | PD23.1_079 | 55180 | 54725 | endonuclease V | 151 | *Enterobacteria* phage T4 | 42.6 | 1e-11 |
| **24** | PD23.1_093 | 66654 | 66190 | translation repressor domain protein | 154 | Viruses | 41.3 | 4e-19 |
| **25** | PD23.1_094 | 67106 | 66684 | putative gp62 clamp loader subunit DNA polymerase accessory protein | 140 | *Dickeya* phage vB_DsoM_LIMEstone1 | 97.9 | 2e-73 |
| **26** | PD23.1_095 | 68100 | 67111 | ATPase associated with various cellular activities family protein | 329 | *Saccharomyces cerevisiae* | 31.9 | 5e-24 |
| **27** | PD23.1_096 | 68848 | 68180 | gp45 sliding clamp, C terminal family protein | 222 | *Enterobacteria* phage RB69 | 25.2 | 3e-07 |
| **28** | PD23.1_097 | 69893 | 69561 | putative uvsW RNA-and DNA-helicase | 110 | Viruses | 47.1 | 4e-13 |
| **29** | PD23.1_100 | 71120 | 70767 | RNA-DNA and DNA-DNA helicase UvsW domain protein | 117 | *Dickeya* phage vB_DsoM_LIMEstone1 | 99.5 | 4e-58 |
| **30** | PD23.1_102 | 72734 | 71988 | PD-(D/E)XK nuclease superfamily protein | 248 | *Dickeya* phage vB_DsoM_LIMEstone1 | 100 | 1e-141 |
| **31** | PD23.1_103 | 73189 | 72734 | recombination, repair and ssDNA binding UvsY family protein | 151 | *Dickeya* phage vB_DsoM_LIMEstone1 | 100 | 9e-83 |
| **32** | PD23.1_104 | 73732 | 73232 | putative gp3-tail completion | 166 | *Dickeya* phage vB_DsoM_LIMEstone1 | 100 | 3e-82 |
| **33** | PD23.1_114 | 80503 | 79175 | major capsid protein | 442 | unclassified *Myoviridae* | 83.9 | 0 |
| **34** | PD23.1_115 | 81467 | 80595 | putative gp22 prohead core protein | 290 | *Dickeya* phage vB_DsoM_LIMEstone1 | 100 | 1e-101 |
| **35** | PD23.1_116 | 82178 | 81513 | prohead core protease family protein | 221 | *Shigella* phage phiSboM-AG3 | 99.1 | 1e-122 |
| **36** | PD23.1_119 | 84402 | 82711 | bacteriophage T4-like capsid assembly family protein | 563 | *Salmonella* phage SKML-39 | 97.3 | 0 |
| **37** | PD23.1_120 | 85003 | 84470 | tail tube protein | 177 | unclassified *Myoviridae* | 87.5 | 2e-81 |
| **38** | PD23.1_122 | 89009 | 87111 | phage tail sheath family protein | 632 | *Enterobacteria* phage T4 | 32.6 | 6e-40 |
| **39** | PD23.1_123 | 91197 | 89062 | pretoxin HINT domain protein | 711 | *Enterobacteria* phage RB49 | 35.9 | 2e-54 |
| **40** | PD23.1_124 | 92137 | 91259 | NUMOD3 motif family protein | 292 | *Dickeya* phage vB_DsoM_LIMEstone1 | 99.3 | 1e-167 |
| **41** | PD23.1_125 | 92816 | 92115 | terminase DNA packaging enzyme family protein | 233 | *Dickeya* phage vB_DsoM_LIMEstone1 | 100 | 1e-117 |
| **42** | PD23.1_126 | 93514 | 92819 | putative gp15 proximal tail sheath stabilization protein | 231 | *Dickeya* phage vB_DsoM_LIMEstone1 | 100 | 1e-134 |
| **43** | PD23.1_127 | 94150 | 93563 | virus neck family protein | 195 | *Dickeya* phage vB_DsoM_LIMEstone1 | 99.1 | 1e-106 |
| **44** | PD23.1_130 | 95990 | 95238 | putative gp13 neck protein | 250 | *Dickeya* phage vB_DsoM_LIMEstone1 | 100 | 1e-132 |
| **45** | PD23.1_134 | 103169 | 101523 | putative tailspike protein | 548 | *Dickeya* phage vB_DsoM_LIMEstone1 | 100 | 0 |
| **46** | PD23.1_135 | 103838 | 103224 | maturation/adhesion domain protein | 204 | *Dickeya* phage vB_DsoM_LIMEstone1 | 100 | 1e-112 |
| **47** | PD23.1_136 | 105403 | 103889 | hemolysin-type calcium-binding domain protein | 504 | *Salmonella* phage SKML-39 | 83.2 | 0 |
| **48** | PD23.1_139 | 109286 | 107508 | baseplate J-like family protein | 592 | *Enterobacteria* phage T4 | 25 | 4e-24 |
| **49** | PD23.1_153 | 118342 | 119775 | DNA polymerase family B, exonuclease domain protein | 477 | *Enterobacteria* phage RB69 | 29.7 | 3e-34 |
| **50** | PD23.1_156 | 120896 | 121375 | DNA polymerase domain protein | 159 | *Dickeya* phage vB_DsoM_LIMEstone1 | 98.7 | 3e-85 |
| **51** | PD23.1_157 | 122251 | 123384 | DNA polymerase B family protein | 377 | *Enterobacteria* phage RB69 | 33.7 | 3e-49 |
| **52** | PD23.1_159 | 123785 | 124564 | 5' nucleotidase, deoxy family protein | 259 | *Dickeya* phage vB_DsoM_LIMEstone1 | 100 | 1e-143 |
| **53** | PD23.1_177 | 132972 | 135728 | putative rIIA protein | 918 | *Escherichia* phage vB_EcoM_CBA120 | 54.6 | 0 |
| **54** | PD23.1_178 | 135760 | 137322 | helix-turn-helix domain protein | 520 | *Salmonella* phage SKML-39 | 93.3 | 0 |
| **55** | PD23.1_182 | 138475 | 139284 | ig-like virion protein | 269 | *Serratia* phage KSP90 | 45.1 | 1e-43 |
| **56** | PD23.1_189 | 143721 | 145052 | DNA gyrase/topoisomerase IV, subunit A family protein | 443 | Schizosaccharomyces pombe 972h- | 30.6 | 6e-49 |
| **57** | PD23.1_192 | 146113 | 146619 | cytidine and deoxycytidylate deaminase zinc-binding region family protein | 168 | *Dickeya* phage vB_DsoM_LIMEstone1 | 100 | 2e-91 |
| **58** | PD23.1_194 | 147798 | 147184 | putative gp4 head completion protein | 204 | *Dickeya* phage vB_DsoM_LIMEstone1 | 100 | 1e-106 |
| **59** | PD23.1_195 | 148535 | 147798 | putative endonuclease segB domain protein | 245 | *Dickeya* phage vB_DsoM_LIMEstone1 | 99.2 | 1e-141 |
| **60** | PD23.1_196 | 148586 | 149554 | putative gp48 T4-like baseplate tail tube cap | 322 | unclassified *Myoviridae* | 97.2 | 0 |
| **61** | PD23.1_198 | 151245 | 151628 | base plate wedge 53 family protein | 127 | *Dickeya* phage vB_DsoM_LIMEstone1 | 98.4 | 3e-67 |
| **62** | PD23.1_199 | 25 | 1395 | RIIA domain protein | 456 | *Shigella* phage phiSboM-AG3 | 92.9 | 0 |
| **63** | PD23.1_200 | 1400 | 1762 | gp44 clamp loader subunit DNA polymerase accessory domain protein | 120 | *Dickeya* phage vB_DsoM_LIMEstone1 | 96.7 | 6e-63 |
| **64** | PD23.1_201 | 1767 | 2189 | putative gp62 clamp loader subunit DNA polymerase accessory protein | 140 | *Dickeya* phage vB_DsoM_LIMEstone1 | 100 | 1e-75 |
| **65** | PD23.1_202 | 2219 | 2683 | translation repressor domain protein | 154 | Viruses | 41.3 | 4e-19 |
| **66** | PD23.1_213 | 9406 | 9804 | loader of gp41 DNA helicase family protein | 132 | *Dickeya* phage vB_DsoM_LIMEstone1 | 98.4 | 1e-67 |
| **67** | PD23.1_217 | 13032 | 12289 | ATP dependent DNA ligase domain protein | 247 | *Dickeya* phage vB_DsoM_LIMEstone1 | 97.6 | 1e-134 |
| **68** | PD23.1_219 | 13813 | 13466 | gp48 T4-like baseplate tail tube cap domain protein | 115 | *Dickeya* phage vB_DsoM_LIMEstone1 | 95.5 | 4e-54 |
| **69** | PD23.1_227 | 17284 | 16775 | DNA gyrase/topoisomerase IV, subunit A family protein | 510 | *Dickeya* phage vB_DsoM_LIMEstone1 | 97.1 | 2e-91 |
| **70** | PD23.1_228 | 17508 | 17305 | gp45 sliding clamp DNA polymerase accessory domain protein | 67 | unclassified *Myoviridae* | 95.4 | 3e-19 |
| **71** | PD23.1_229 | 17960 | 18478 | endoribonuclease RegB T4-bacteriophage encoded family protein | 172 | *Dickeya* phage vB_DsoM_LIMEstone1 | 96.6 | 6e-83 |
| **72** | PD23.1_230 | 18487 | 18837 | RIIB domain protein | 116 | *Dickeya* phage vB_DsoM_LIMEstone1 | 93 | 1e-56 |
| **73** | PD23.1_231 | 18842 | 19198 | RIIA domain protein | 118 | *Dickeya* phage vB_DsoM_LIMEstone1 | 94.9 | 3e-56 |
| **74** | PD23.1_234 | 24112 | 22466 | putative tailspike protein | 548 | *Enterobacteria* phage K1F | 49.1 | 4e-06 |
| **75** | PD23.1_235 | 27044 | 24168 | putative tailspike protein | 958 | *Salmonella* phage SKML-39 | 52 | 3e-88 |
| **76** | PD23.1_241 | 33429 | 33578 | major capsid domain protein | 49 | unclassified *Myoviridae* | 83 | 1e-16 |
| **77** | PD23.1_242 | 33680 | 34528 | NUMOD3 motif family protein | 282 | Viruses | 64.4 | 1e-114 |
| **78** | PD23.1_245 | 35238 | 35648 | putative phage associated protein | 136 | unclassified *Myoviridae* | 97.8 | 2e-71 |
| **79** | PD23.1_247 | 36244 | 36035 | uvsX RecA-like recombination domain protein | 69 | *Dickeya* phage vB_DsoM_LIMEstone1 | 97 | 2e-31 |
| **80** | PD23.1_248 | 37122 | 36709 | ATPase associated with various cellular activities family protein | 137 | *Saccharomyces cerevisiae* | 36 | 3e-14 |
| **81** | PD23.1_250 | 37135 | 37374 | topoisomerase II large subunit domain protein | 79 | *Salmonella* phage SKML-39 | 94.8 | 2e-26 |
| **82** | PD23.1_251 | 37383 | 37955 | AP2 domain protein | 190 | *Dickeya* phage vB_DsoM_LIMEstone1 | 100 | 1e-112 |
| **83** | PD23.1_252 | 37952 | 38158 | DNA gyrase/topoisomerase IV, subunit A family protein | 68 | *Dickeya* phage vB_DsoM_LIMEstone1 | 97 | 2e-27 |
| **84** | PD23.1_254 | 38628 | 39260 | putative gp13 neck protein | 210 | *Salmonella* phage SKML-39 | 94.7 | 1e-102 |
| **85** | PD23.1_256 | 39560 | 40069 | virus neck family protein | 169 | unclassified *Myoviridae* | 97.6 | 1e-91 |
| **86** | PD23.1_260 | 41401 | 40838 | gp5 baseplate hub subunit and tail lysozyme domain protein | 187 | *Enterobacteria* phage T4 | 78 | 4e-10 |
| **87** | PD23.1_268 | 45889 | 45191 | gp61 DNA primase subunit domain protein | 232 | *Dickeya* phage vB_DsoM_LIMEstone1 | 99.2 | 1e-130 |
| **88** | PD23.1_269 | 45920 | 46462 | dUTPase family protein | 180 | *Dickeya* phage vB_DsoM_LIMEstone1 | 92.1 | 2e-93 |

^a^ – predicted function is based on amino acid sequence identity, presence of conserved motives, gene location in the functional modules

^b^ - protein length (number of amino acids)
